# Feasibility of quantifying change in immune white cells in abdominal adipose tissue in response to an immune modulator in clinical obesity

**DOI:** 10.1371/journal.pone.0237496

**Published:** 2020-09-03

**Authors:** Fred R. Sattler, Melissa Mert, Ishwarya Sankaranarayanan, Wendy J. Mack, Lauriane Galle-Treger, Evelyn Gonzalez, Lilit Baronikian, Kyuwan Lee, Pedram Shafiei Jahani, Howard N. Hodis, Christina Dieli-Conwright, Omid Akbari

**Affiliations:** 1 Department of Medicine, University of Southern California Keck School of Medicine, Los Angeles, California, United States of America; 2 Department of Preventive Medicine, University of Southern California Keck School of Medicine, Los Angeles, California, United States of America; 3 Department of Molecular Microbiology and Immunology, University of Southern California Keck School of Medicine, Los Angeles, California, United States of America; 4 Ostrow School of Dentistry, Division of Physical Therapy and Biokinesiology, University of Southern California, Los Angeles, California, United States of America; 5 Department of Population Sciences, Beckman Research Institute, City of Hope National Medical Center, Duarte, California, United States of America; Università degli Studi di Milano, ITALY

## Abstract

**Background:**

Obesity is often associated with inflammation in adipose tissue (AT) with release of mediators of atherogenesis. We postulated that it would be feasible to collect sufficient abdominal AT to quantify changes in a broad array of adaptive and innate mononuclear white cells in obese non-diabetic adults in response to a dipeptidyl protease inhibitor (DPP4i), known to inhibit activation of immune white cells.

**Methods:**

Adults 18–55 years-of-age were screened for abdominal obesity and insulin resistance or impaired glucose tolerance but without known inflammatory conditions. Twenty-one eligible participants consented for study and were randomized 3:1 to receive sitagliptin (DPP4i) at 100mg or matching placebo daily for 28 days. Abdominal AT collected by percutaneous biopsy and peripheral blood mononuclear cell fractions were evaluated before and after treatment; plasma was stored for batch testing.

**Results:**

Highly sensitive C-reactive protein, a global marker of inflammation, was not elevated in the study population. Innate lymphoid cells (ILC) type 3 (ILC-3) in abdominal AT decreased with active treatment compared with placebo (p = 0.04). Other immune white cells in AT and peripheral blood mononuclear cell (PBMC) fractions did not change with treatment compared to placebo (p>0.05); although ILC-2 declined in PBMCs (p = 0.007) in the sitagliptin treatment group. Two circulating biomarkers of atherogenesis, interferon-inducible protein-10 (IP-10) and sCD40L declined in plasma (p = 0.02 and p = 0.07, respectively) in the active treatment group, providing indirect validation of a net reduction in inflammation.

**Conclusions:**

In this pilot study, two cell types of the innate lymphoid system, ILC-3 in AT and ILC-2 PBMCs declined during treatment and as did circulating biomarkers of atherogenesis. Changes in other immune cells were not demonstrable. The study showed that sufficient abdominal AT could be obtained to quantify white cells of both innate and adaptive immunity and to demonstrate changes during therapy with an immune inhibitor.

**Trial registration:**

ClinicalTrials.gov identifier (NCT number): NCT02576

## Background

Abdominal obesity is a central component of the metabolic or insulin resistance syndrome that is associated with an increased risk for heart attack, stroke and peripheral vascular disease [1]. The mechanisms as to how abdominal adipose tissue (AT) contributes to these complications in humans is uncertain. In laboratory models of obesity, adipocytes progressively accumulate lipid. As these cells increase in size and cause local tissue anoxia and cell death, AT converts from an anti-inflammatory state with predominantly M2 macrophages to an inflammatory phenotype with influx of M1 inflammatory monocytes that become resident tissue inflammatory macrophages (also designated M1) that secrete chemokines and pro-inflammatory cytokines [2–4]. Secretion of these mediators impairs insulin signaling and together with the efflux of pro-atherogenic mediators to the systemic circulation induce events that presumably damage the vascular endothelium and cause endothelial dysfunction, early manifestations of atherosclerosis.

In humans, abdominal obesity is associated with insulin resistance, circulation of inflammatory cytokines (e.g. IL-6, TNFα) [5, 6], other mediators such as chemokines and vascular adhesion molecules that are together central to the pathogenesis of endothelial dysfunction and eventually atherosclerotic plaque [7]. These clinical abnormalities become more apparent with aging [5, 8]. It is unclear why many adults with abdominal obesity show this clinical phenotype of “unhealthy” obesity [3, 4] whereas other abdominally obese persons do not manifest these abnormalities and have “healthy” obesity [8].

Traditional methods have examined AT for the presence of crown-like structures (CLS) which represent dying adipocytes that are surrounded by inflammatory macrophages (M1), a signature for AT inflammation. These structures are limited in numbers and minimally present in non-inflamed AT where the resident macrophages are predominantly anti-inflammatory (M2) cells. However, enumeration of M1 and M2 macrophages and their regulatory T cell fractions (effector and regulatory cells) requires sizeable quantifies of AT.

Innate lymphoid cells (ILCs) are integral in maintaining tissue microstructure and may also be important in the regulation of obesity and metabolism [9–11]. These cells are characterized by their cytokine production such that ILC-1 and ILC-3 are primarily inflammatory and ILC-2 anti-inflammatory [12, 13] and thus may interact with monocytes/macrophage and T-lymphocytes. Early evidence suggests that ILCs may have a role in the pathogenesis of obesity and dysmetabolism. For instance, ILC-1 may foster insulin resistance even without affecting total body weight in mouse models [14, 15]. Whereas, ILC-2 isolated from murine and human white adipose tissue (WAT) when activated can promote beiging of WAT [9] and induction of uncoupling protein 1 (UCP1) to limit the development of obesity [9–11]. The role of ILC-3 in obesity is less clear. Cytokines produced by ILC-3 including the lymphotoxin/IL-23/IL-22 pathway may promote induction of obesity [16]; however, IL-22 may be protective against the development of obesity [17, 18]. To study these potentially important immune white cells in obesity, substantial amounts of tissue are needed to quantify the ILCs and their functional importance since they are present in much lower numbers than other immune white cells.

To date, obtaining sufficient amounts of intact abdominal adipose tissue (AT) to study histology and quantify a broad array of immune white blood cells has not been possible. Liposuction can provide a large slurry of adiposity but the liquid collection cannot be used to study AT histology or to enumerate CLS signifying an inflammatory phenotype [5, 6] or for special histochemical staining. More traditional biopsies of subcutaneous AT can yield intact tissue but quantifies are often limited to 1-to-2g or less, which is insufficient for both histologic examination and comprehensive quantification of different immune cells. We reported that much more sizeable quantities of intra-abdominal AT can be obtained using a novel methodology [19]. We have since refined the method to consistently acquire up to 10g of abdominal AT, which should be sufficient for comprehensive immune cell phenotyping using fluorescence activated cell sorting (FACS).

In this study, we determined whether sufficient amounts of abdominal AT could be obtained with our methodology to quantify changes in numbers of M1 and M2 monocytes/macrophages, Teffector and Tregulatory cells and innate lymphoid cells (ILC-1, ILC-2 and ILC-3) by FACS in response to a short course of therapy with a dipeptidyl dipeptidase 4 (DDP4) inhibitor, sitagliptin. Sitagliptin is a weak oral hypoglycemic agent that increases incretin levels to lower blood glucose levels [20, 21]. The drug is of immunological interest since it suppresses monocyte/macrophage signal transduction and activation [22–24], cells which may be further regulated and counter regulated by T-lymphocytes. There are no reported clinical data on the effects of DPP4 inhibition of white cells of the innate immune response, which we postulated could also be important in regulation of abdominal adipose tissue inflammation [25–27]. We elected to use sitagliptin as a probe to assess whether we could detect change in numbers of different white cell phenotypes of adaptive and innate immunity in obese humans. We report herein that our approach for AT acquisition and FACS can be used to quantify changes in white cells of both arms of immunity in abdominal obesity. Secondarily, we also determined whether any change in AT cellular immune cell counts could be related to changes in several important plasma mediators and biomarkers that reflect increased risk for cardiovascular disease.

## Methods

The study reported in this manuscript was approved by the Institutional Review Board of the University of Southern California (approval #HS-13-00345). All study participants including those screened for enrollment signed written informed consent before any measurements or interventions in the protocol were initiated.

### Hypothesis and study design

We postulated that it would be feasible to collect sufficient abdominal AT to quantify changes in a broad array of adaptive and innate mononuclear white cells in obese non-diabetic adults in response to treatment with a dipeptidyl protease inhibitor (DPP4i), known to inhibit immune white cell activation. We secondarily sought to determine whether this treatment could be related to reductions in plasma biomarkers of atherogenesis.

This pilot study included a placebo-treated control group to qualitatively assess whether there may be sizable changes over time without treatment (including possibly regression to the mean), although the sample size was generally underpowered to demonstrate statistical significance between the groups. Eligible participants were randomized 3:1 by computer generated blocks of four to receive treatment with sitagliptin as a single 100mg tablet versus matching placebo taken once daily for 28 consecutive days.

To be eligible, participants had to be 18–55 years of age and have abdominal obesity based on minimum waist circumference (minimum visual waist between lower rib margins and upper iliac crest) of at least 95cm for men and 94cm for women when measured in triplicate. These break points for obesity have been validated with increases in visceral adipose tissue [28] related to cardiometabolic risks [29, 30]. Participants also had to have evidence of impaired glucose tolerance or insulin resistance based on fasting blood glucose of 100–125 mg/dL (5.55–6.94 mmol/L), Hgb A1C of 5.7 to 6.4% or HOMA-IR of ≥3.0 with no history of or current treatment for diabetes. Potential participants were excluded if they were pregnant or lactating; had clinical evidence of cardiovascular disease, an active inflammatory condition, hepatic or renal disease; or regularly used anti-inflammatory or statin drugs.

### Abdominal fat biopsies

Participants were instructed to fast after 10PM and were admitted the following morning to the inpatient USC Clinical Trials Unit (CTU). Biopsies were performed as previously described [19]). In brief, the abdominal skin was prepared with three betadine scrubs at the biopsy site in the right anterior axillary line at the level of the umbilicus. After infiltration of the dermis and very superficial subcutaneous tissue with 1% lidocaine, a 6–7 mm incision was made in the skin with #11 Bard Parker scalpel. A 6-mm Bergström side-cutting needle (Micrins Surgical, Lake Forest, IL, USA) was introduced approximately 1–1.5 inches through the incision into the subcutaneous abdominal AT. Suction was then applied from a 60-cc syringe attached by irrigation tubing to the Bergström needle. Four cuts were made with the cutting trochar as the needle was further advanced and rotated 90 degrees prior to each cut. The procedure consistently yielded 1.5-8g of adipose tissue. Manual compression was applied to the wound and the incision was closed with 3.0 silk suture using a figure-of-8 tie. The participant was discharged from the CTU with instructions for post procedure wound care.

### Flow cytometry: Adipose tissue and blood

**Fig 1:**
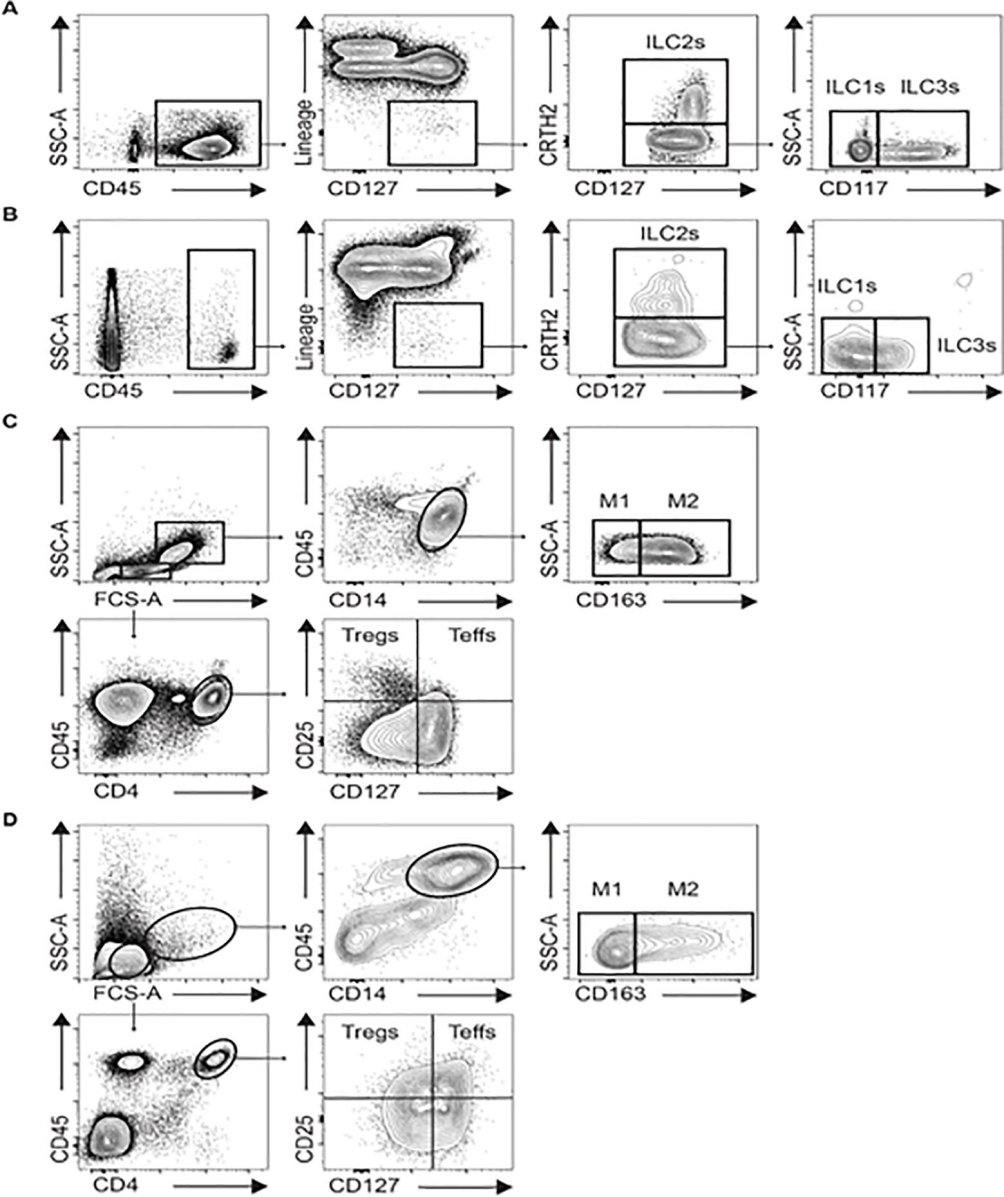
Leukocyte gating strategies in human blood and adipose tissue. Gating strategy of IL-1, ILC-2 and ILC-3 cells in blood (A) and in adipose tissue (B). Total ILCs were identified as CD45^+^ cells negative for lineage markers and positive for CD127. ILC-2s are CRTH2, whereas ILC-1s and ILC-3s are defined as CRTH2^**-**^ CD117^**-**^ and CRTH2^-^ CD117^**+**^ cells, respectively. Gating strategy of Tregs, Teffs, M1 and M2 cells in blood (C) and in adipose tissue (D). Tregs and Teffs are both positive for CD45, CD4 and CD25. Additionally, while Tregs are CD127^-^, Teffs are CD127^**+**^. M1 and M2 cells are gated as positive for CD45 and CD14. M1 cells are identified as CD163^**-**^ whereas M2 cells are CD163^**+**^.

Inflammatory and anti-inflammatory cells were measured in AT and peripheral blood mononuclear cells (PBMCs) by flow cytometry. The immune cell populations quantified and isolated included M1 and M2 macrophages and monocytes, T regulatory and T effector lymphocytes and the three innate lymphoid cell subsets (ILC-1, ILC-2 and ILC-3). PBMCs were first isolated from whole blood by diluting the blood 1:1 in PBS and adding to SepMate^TM^-50 separation tubes (STEMCELL Technologies Inc, Vancouver, Canada) prefilled with 15mL Lymphoprep^TM^ each (Axis-Shield, Oslo, Norway) and centrifugated at 1200g for 15 minutes. Subcutaneous adipose tissue samples were weighed and digested in collagenase IV (200U/mL, Worthington Biochemical Corporation) at 37°C for one hour and then processed on a 70μm nylon cell strainer (Falcon®) into a single cell suspension. The stromal vascular fraction from the subcutaneous adipose tissue and the blood mononuclear cells were stained with the following surface marker antibodies: CD4, CD45, CD25, CD163, CD127, CD14, Lineage (CD3, CD14, CD16, CD19, CD20, CD56) CD235a, FCεRIα, CD1a, CD123, CD161, CRTH2, CD117; ThermoFisher scientific). The following established gating strategies based on previous publications [31–35] were used to quantify the different immune cell sub-populations using the eight-color BD FACSCANTO analyzer and data were acquired using BD FACSDiva software (BD Bioscience, San Jose, CA): CD45^+^, CD14^+^, CD163^-^ for M1 macrophages, CD45^+^, CD14^+^, CD163^+^ for M2 macrophages, CD45^+^, CD4^+^, CD25^+^, CD127^low^ for regulatory T cells, CD45^+^, CD4^+^, CD25^+^, CD127^high^ for T effector cells. CD45^+^, Lineage^-^, CD127^+^, CRTH2^-^, CD117^-^ for ILC1s, CD45^+^, Lineage^-^, CD127^+^, CRTH2^+^ for ILC2s, CD45^+^, Lineage^-^, CD127^+^, CRTH2^-^, CD117^+^ for ILC3s [35]. The total number of M1, M2, Tregs, Teff, ILC-1, ILC-2, and ILC-3 subsets were quantified using FlowJo version 10 software (TreeStar Ashland OR) and were normalized to grams of AT collected or per milliliter of blood collected, respectively, as previously described [25, 33, 36, 37]. Gating strategy and flow cytometry of a representative sample are shown in Fig 1.

### Participant recruitment and follow up dates

Potential study participants were screened for study eligibility after signing a University of Southern California Institutional Review Board approved informed consent beginning February 15, 2016; the final subject was screened on February 27, 2016. The last participant had final clinical and laboratory measurements on April 28, 2017.

### Clinical outcomes

#### Plasma glucose and soluble biomarkers

Blood was collected in EDTA phlebotomy tubes for plasma glucose levels and batch testing for biomarkers. One tube was placed immediately in an ice bath and within 15–20 minutes plasma was separated after spinning at 1500g for 15min in a 4C refrigerated centrifuge. Samples were immediately tested in triplicate on a glucose analyzer (Yellow Springs Instruments, Model 2300; coefficient of variation <2%) calibrated with glucose standards in the prior 1 hour. The average of the three sample runs was used for eligibility screening. The remainder of the plasma was processed in a similar manner and stored at -80C.

Frozen samples of stored plasma were thawed once and batched tested for circulating biomarker levels including plasma C-Reactive Protein ([CRP] with coefficient of variation (CV) = 3.8–8.3%, sensitivity = 0.022ng/ml), hsIL-6 (CV = 6.9–7.8%, sensitivity = 0.11pg/mL), hsTFNα (CV = 3.1–7.7%, sensitivity = 0.106pg/mL), sCD26 (CV = 4.0–7.3%, sensitivity = 0.072pg/mL), sCD40L (CV = 4.5–5.4%, sensitivity = 10.0pg/mL) and IP-10 (CV = 3.0–4.6%, sensitivity = 4.46pg/mL) before and after 28 days of treatment with sitagliptin using reagents from R&D Systems Inc.

#### Other outcomes

Complete blood counts and comprehensive chemistries were obtained on study participants before, during and at completion of study therapy to monitor for safety. Methods for vascular imaging (brachial artery flow mediated dilation and carotid artery intima media thickness) are described in the complete study protocol (S1 File).

### Statistical analyses

Differences in baseline characteristics and measurements by randomized treatment group were analyzed by Fisher’s exact test or exact Wilcoxon rank-sum test, as appropriate. Results are reported as proportions for categorical variables and both means and standard deviations and medians and interquartile ranges (IQRs) are reported for continuous variables. Differences in change (day 28 minus baseline) in assay measurements between treatment groups were compared by ANCOVA, using a rank transformation, controlling for baseline of the measurement. Results are reported as mean (95% confidence interval) and median (interquartile range). Within-group changes from baseline were assessed by Wilcoxon signed-rank test. All statistical analyses were performed using SAS version 9.4.

Pass Software [38] was used to determine the minimum detectable change in an outcome with the planned sample size of 20 subjects randomized 3:1 to receive sitagliptin versus matching placebo. Assuming up to 10% attrition (i.e. 18 participants completing the study), alpha = 0.05(two-sided) and 80% power, we would be able to statistically detect a large between group differences in pre-post changes (Cohen’s d = 1.7), assuming a moderate within participant correlation in pre-posttest measurements (r = 0.5). For the group receiving sitagliptin, we would be able to detect a within group effect size on change of at least d = 0.81.

## Results

### Study flow and participant characteristics

**Fig 2:**
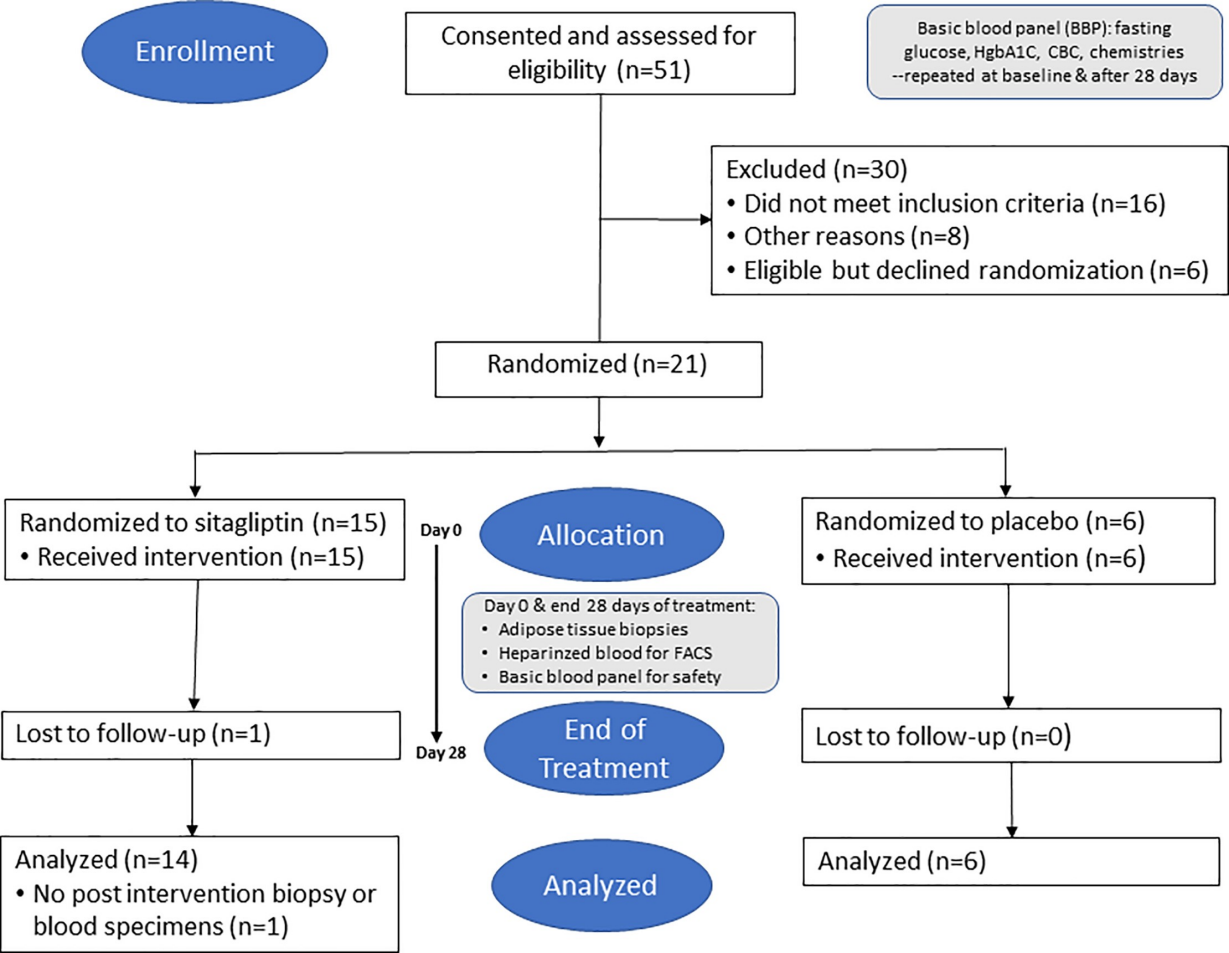
Study flow chart.

Fifty-one potential study participants signed informed consistent approved by the USC institutional Review Board and were screened for eligibility. Lack of impaired glucose tolerance or insulin resistance (n = 10), lack of abdominal obesity defined for eligibility (n = 3) and presence of diabetes (n = 3) were the most common reasons for exclusion. Four were excluded for individual reasons, and after initial screening four other potential participants elected not to undergo further testing. Twenty-seven participants were fully eligible; of these six decided not to participate generally because of scheduling conflicts. Twenty-one eligible participants were randomly assigned to receive blinded treatment with sitagliptin (n = 15) or matching placebo (n = 6; Fig 2).

The two groups were quite comparable (Table 1). Of note, plasma C-reactive protein (CRP) was well below the upper limit of normal of 3 mg/dL for the cohort of 21 participants. One participant assigned to placebo moved away midway during treatment and did not return for follow up. Twenty participants completed all aspects of the study including end of treatment abdominal fat biopsies and serve as the basis for these analyses.

**Table 1:** Patient characteristics prior to treatment.

	Sitagliptin	Placebo	P-value
	n = 15	n = 6	
Female	10 (66.7%)	2 (33.3%)	
Male	5 (33.3%)	4 (66.7%)	0.33
African American	2 (13.3%)	0	
Caucasian	1 (6.7%)	1 (16.7%)	1.00
Other (Latino)	12 (80.0%)	5 (83.3%)	
Hispanic ethnicity	12 (80.0%)	5 (83.3%)	1.00
Age, years	38.8 ± 9.3 ^#a^	38.8 ± 9.1	0.89
	38 (32–47) ^#b^	36 (32–49)	
Weight, kg	101.1 ± 16.9	114.3 ± 21.5	0.21
	100.9 (84.8–109.2)	109.8 (99.0–137.3)	
Min waist circumference, in	43.0 ± 6.1	46.6 ± 4.8	0.18
	41.0 (39.7–46.5)	46.5 (42.7–50.8)	
Iliac waist circumference, in	46.6 ± 5.3	48.9 ± 6.2	0.65
	46.3 (42.2–48.2)	49.8 (43.0–54.8)	
Glucose, mg/dL^#c^	100.9 ± 10.6	103.3 ± 9.9	0.51
	98.5 (94.0–103)	106 (93.5–109)	
HOMA-IR	5.3 ± 2.1	5.0 ± 3.2	0.40
	5.1 (3.9–6.1)	3.7 (3.5–5.3)	
HgbA1C, %	5.6 ± 0.5	5.9 ± 0.2	0.06
	5.5 (5.3–5.8)	5.9 (5.8–6.0)	
C-reactive protein, mg/dL^#d^	1.067 ± 0.930	0.558 ± 0.569	0.24
	0.757 (0.318–1.9442)	0.435 (0.195–0.663)	

^a#^ Geometric mean and one standard deviation

^#b^ Median (interquartile range)

^#c^ mmol/L glucose is equal to mg/dL glucose X 0.0555

^d#^ nmol/L C-reactive protein is equal to mg/dL C-reactive protein X 9.524

### Fluorescent activated cell sorting

**Fig 3:**
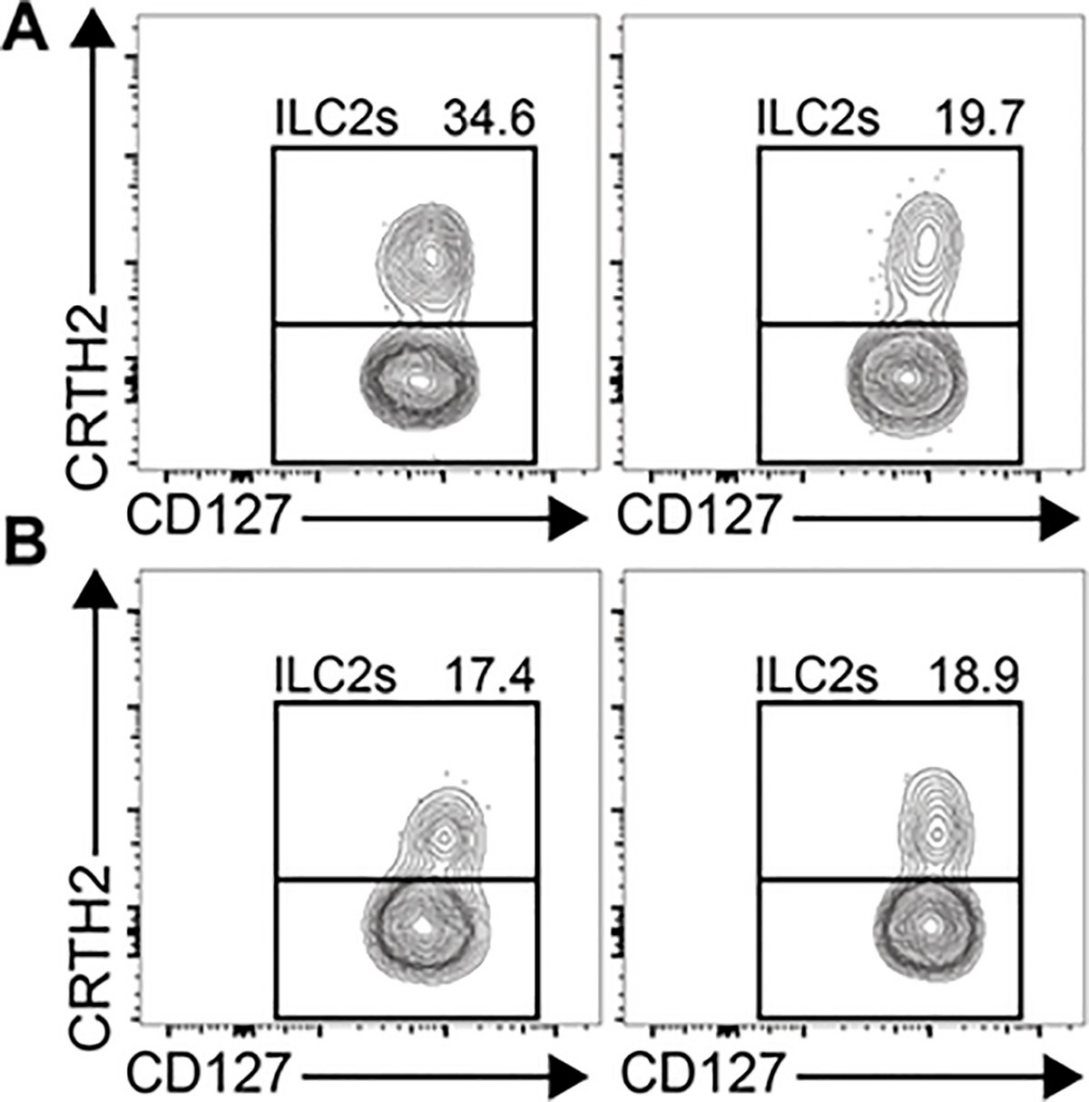
ILC3s decrease in adipose tissue but ILC2s remain the same. ILC-3 numbers/gram of adipose tissue decreased significantly in participants treated with sitagliptin compared to placebo (p = 0.03). Panel A -shows an example of the decrease in ILC-3 in a participant before and after treatment with sitagliptin, respectively. In the same participant, panel B shows no difference in ILC-2 before and after treatment, respectively.

**Fig 4:**
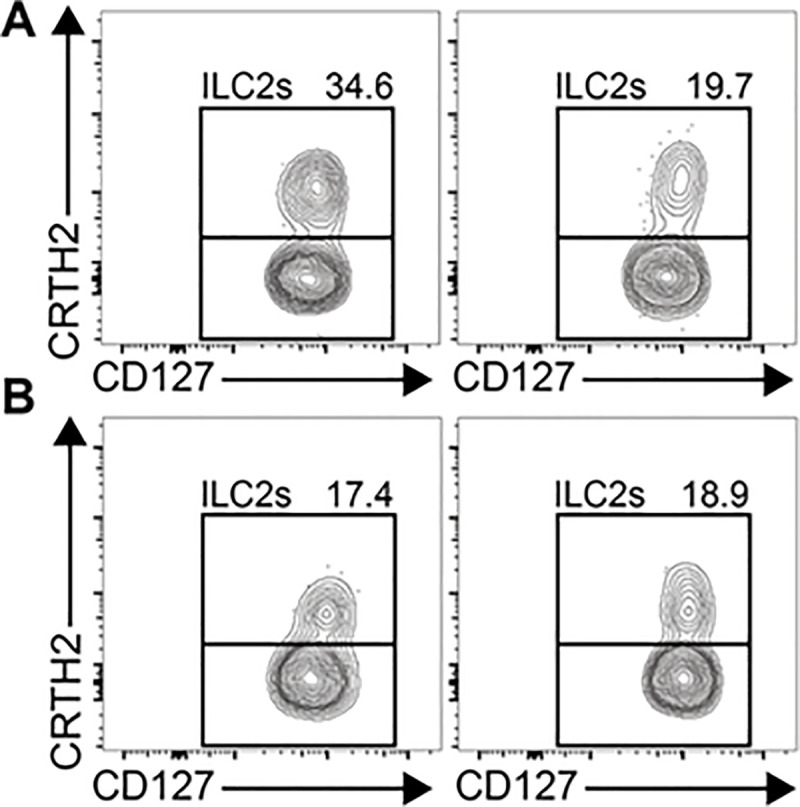
ILC2s decrease in blood but remain the same in adipose tissue. In the sitagliptin-treated participants, changes in the ILCs in peripheral blood mononuclear cell (PBMC) fractions often were not congruent with the change or lack of change in the same cell lines in abdominal adipose tissue (data not shown). Panel A exemplifies a decrease in ILC-2 in PBMCs from a participant before and after 28 days of treatment with sitagliptin, respectively. Panels B shows no difference in ILC-2 from adipose tissue in the same participant before and after treatment, respectively.

All 20 participants provided sufficient AT samples at both baseline (median [range]: 3.8 [1.9–5.5] g) and follow-up (median [range]: 3.6 [1.6–4.6] g) for the predetermined testing. Use of FACS was, therefore, able to accurately discriminate change and lack of changes in ILC subgroups for individual patients as exemplified for a participant as shown in Fig 2. Moreover, the changes of different ILC subgroups with treatment in adipose tissue was not the same -as in PBMCs (Table 2) and exemplified by a patient in Fig 3. At 6 biopsy sessions, 8-to-10g of AT was obtained with more biopsy passes and cuts and would have been sufficient if resources had been available for histologic characterization and other immune markers (Fig 4).

**Table 2:** Changes in metabolism and innate lymphoid cells after 28 days of treatment with sitagliptin.

Variable	Baseline	Day 28	Change	P-value^#b^
***In plasma***				
ALT, *units/L*	28 (14–34) ^#a^	23 (16–45)	-3 (-9–0)	0.08
Fasting glucose *mg/dL* ^#c^	98.3 (93.8–103)	89.0 (86.0–97.0)	-6.00 (-8.80 –-1.80)	<0.001
HgbA1C *%*	5.5 (5.3–5.8)	5.5 (5.4–5.7)	-0.1 (-0.2–0)	0.02
Insulin, *μIU/ml*	13.7 (8.20–20.1)	15.4 (8.49–22.34)	-0.17 (-3.18–2.76)	0.76
HOMA-IR	3.46 (2.03–6.31)	3.51 (1.74–5.98)	-0.34 (-0.71–0.39)	0.36
***Innate lymphoid cells*** *(absolute counts)*				
In adipose tissue cells/g				
ILC-1 cells	320 (102–820)	194 (59–386)	-206 (-810 –-83)	0.06
ILC-2 cells	264 (28–338)	98 (11–176)	-19 (-268–5)	0.31
ILC-3 cells	42 (10–48)	25 (14–37)	-3 (-28–17)	1.00
In blood cells/ml				
ILC-1 cells	303 (104–644)	189 (90–539)	-116 (-162–70)	0.08
ILC-2 cells	139 (79–210)	78 (39–140)	-31 (-91– -8)	0.007
ILC-3 cells	63 (32–108)	30 (14–154)	-9 (-57–13)	0.58

^#a^ Values are expressed as median (interquartile range) for baseline, day 28, and change (day 28—baseline) for all variables.

^#b^ P-values were obtained by Wilcoxon signed rank test on change.

^#c^ Mmo/L glucose is equal to mg/dL glucose X 0.0555.

### Between group comparisons

After adjusting for baseline levels of ILC-3, ILC-3 in AT decreased by 4.0 (95% CI: -43.6, 35.5) cells/g of tissue after treatment with sitagliptin compared to an average increase of 85.2 (95% CI: 18.9, 151.4) cells/g of tissue observed in the placebo group (ANOVA on ranks, p = 0.04). The median change was -3.3 (IQR -27.7, -16.9) cells/g for the sitagliptin group and 74.5 (IQR -1.1, -116.7) cells/g for the placebo group, without accounting for differences in baseline. There were no other significant changes in absolute numbers of immune cells between treatment groups. S1 and S2 Figs show representative dot plots of CD45+ cells, Tregs, Teff, M1 and M2 in the stromal fraction of AT and PBMCs immediately before and after 28 days of study treatment, which is consistent with a lack of change in distribution of immune cells as well as their absolute numbers.

### Within sitagliptin-group change

For participants who received sitagliptin, ILC-1 cells decreased in AT by 206 cells/g (IQR of decrease 810 to -83, p = 0.06); in PBMCs, ILC-1 cells decreased by 116 cells/ml (IQR of decrease 162 to 71, p = 0.08) and circulating ILC-2 cells declined by 31 cells/ml (IQR of decrease 91 to 8.5, p = 0.007 (Table 2 For the other immune cells (Tregs, Teffs, M1, and M2) along with total CD45+ cells in AT stromal fractions and PBMCs, there were no differences in absolute cell counts or their relative percentages immediately before or after 28 days of treatment with sitagliptin (S1 Table). This data further validates that the distribution of different immune white cells was not affected by active treatment with sitagliptin.

Fasting plasma insulin and HOMA-IR did not change significantly (Table 2). On average, fasting glucose levels declined by 6.0mg/dL (IQR of decline 8.8 to -1.8) (p<0.001) and HgbA1C levels declined by 0.10% (IQR of decline 0.20% to– 0%, p = 0.02) after 28 days of sitagliptin treatment. Change in measures of glycemia (fasting glucose, insulin, HOMA-IR and HgbA1C) and change in ILCs in AT and PBMCs were not related (p>0.15) with one exception. Reductions in HgbA1C was positively related to reduction of ILC-2 (Spearman r = 0.50) in circulating PBMCs but the relationship did not reach significance (p = 0.07, S2 Table), The relationship between these two parameters in AT was less pronounced (Spearman r = 0.39, p = 0.19).

**Fig 5:**
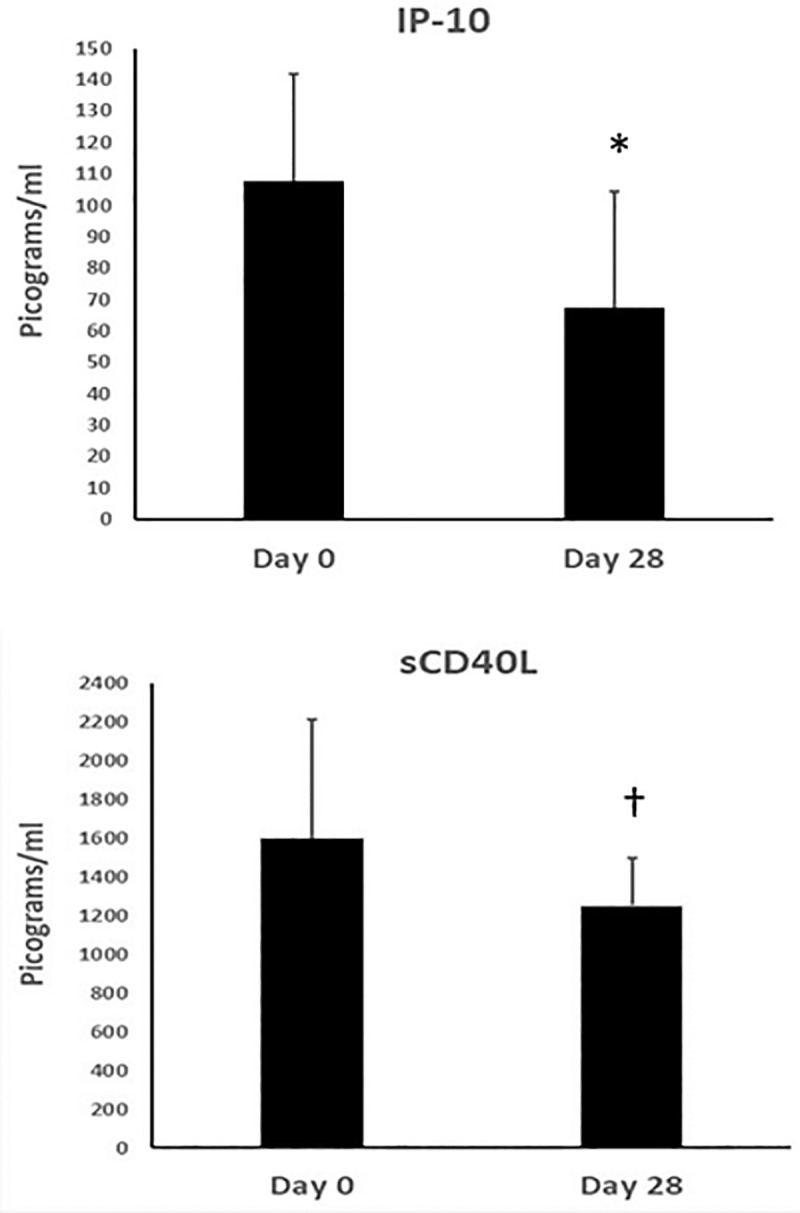
Change in plasma biomarkers of cardiovascular disease after 28-days of treatment with sitagliptin. Data represent median values with whiskers up to the 3^rd^ quartile for soluble interferon gamma inducible protein (IP-10) and soluble CD40 ligand (sCD40L) at baseline (day 0) and after 28 days of treatment with sitagliptin. For IP-10, differences after 28 days were significant at p = 0.02 (*) and for sCD40L the change did not reach significance p = 0.07 (†) by Wilcoxon signed-rank test.

Two plasma biomarkers declined: IP-10 declined by 30.5 units/ml (IQR decline of 59.9 to 14.29, p = 0.02) and sCD40L declined by 285 unit/ml (IQR decline of 1231 to 149.5, p = 0.07; Fig 5). However, CRP, hsIL-6 and hsTNFγ did not change (data not shown). The change in sCD26, which is released from activated immune white cells was directly correlated with change in numbers of ILC-2 cells in AT but the change did not reach significance (rho = 0.49, p = 0.09; data not shown).

### Other outcomes

There were no serious clinical or laboratory adverse events associated with study therapy or the biopsy procedure. There were no significant pre to post changes between or within groups (p>0.10) for the vascular imaging outcomes (data not shown).

## Discussion

We postulated that it would be possible to collect sufficient abdominal AT to quantify changes in a broad array of adaptive and innate mononuclear white cells in obese patients in response to treatment with an immune modulator known to inhibit activation of immune white cells. This hypothesis was supported as we demonstrated changes in various ILCs in AT and PBMCs. However, there was no change in M1 and M2 macrophages and monocytes or T-regulatory and T-effector lymphocytes. The discordant findings between the change in ILCs of the innate immune response and lack of change in the more traditional white cells of the adaptive immune response is unclear. However, the fact that unexpectedly our population lacked evidence of systemic inflammation based on normal CRP prior to treatment may explain the lack of change in the traditional immune white cells. Reductions in circulating IP-10 and sCD40Lvalidate that alterations in ILCs in AT were occurring in response to treatment.

Further, the moderately strong positive correlation of change in plasma sCD26 with change in AT tissue ILC-2 (r = 0.49, that did not reach significance, p = 0.09) supports the expected effects of sitagliptin on the DPP4 enzyme activity that resides in the cellular receptor CD26 present on ILC-2 cells [39]. Thus, DPP4i may also have directly attenuated anti-inflammatory activity of ILC-2s, possibly as counter regulation from suppression of inflammatory ILC-1 and ILC-3. Together these results suggest that DPP4 inhibition with sitagliptin may have globally attenuated immune activation in AT. Finally, the observation that improvement in glycemic control (reductions in HgbA1C) was positively correlated with reductions of ILC-2 in PBMC (not AT, r = 0.50, p = 0.07) is also consistent with an unobserved global reduction in inflammation, which impairs insulin signaling, and that ILC-2 counts declined secondarily. Regardless, the fact that changes in PBMC cell types were not concordant with changes in AT (namely ILC-1 and ILC-3 declined in AT and ILC-2 fell in PBMCs) emphasizes the importance of tissue sampling to assess effects of treatment interventions on these cell types.

Decline of two soluble proatherogenic mediators and markers of atherogenesis, IP-10 and sCD40L during treatment with sitagliptin suggests that there was a net down regulation in inflammation. Interferon inducible protein 10 (IP-10) is a chemokine secreted by monocytes, macrophages and endothelial cells in response to IFNγ [40], which is secreted by ILC-1 cells [25]. Further, IP-10 secreted by these immune cells binds to the G-receptor of endothelial cells, which contribute to the pathogenesis of arterial plaque [41–44]. Levels of plasma IP-10 and/or its receptor (CXCR3) are increased in a number of cardiovascular disorders [41, 42, 45–47]. Similarly, sCD40L also declined during treatment, although the change did not reach traditional significance (p = 0.07), the decrease supports the effects of sitagliptin on IP-10. Soluble CD40 ligand is shed from activated M1 monocytes and macrophages and binds the Type I CD40 transmembrane receptors of endothelial and smooth muscle cells [48, 49] as well as macrophages and monocytes in the vasculature and arterial plaque [50, 51]. This ligation promotes secretion of a broad range of chemokines, inflammatory cytokines and vascular adhesion molecules that are central to the pathogenesis of atherosclerosis. Plasma levels of sCD40L are increased in persons with fat inflammation, at risk for type 2 diabetes, with arterial plaque instability, and acute myocardial infarction [47, 52–55]. Change in these systemic biomarkers of atherogenesis that occurred concordant with changes in ILCs emphasizes the importance of quantifying all immune cells in understanding pathogenesis of obesity-related risk for cardiovascular disease.

The changes in immune ILCs occurred in the absence of any statistically significant effects on traditional biomarkers of inflammation (e.g. soluble IL6 and TNFα) or the marker of global inflammation, CRP. This was not surprising since prior to treatment with sitagliptin, plasma CRP levels were well within the normal range for the study participants. The normal CRP was unexpected since a substantial portion of abdominally obese persons demonstrate systemic inflammation [6, 7]. However, the fact that DPR4i attenuated inflammatory ILC-3 cells in AT in the between group analysis (p = 0.04) and AT inflammatory ILC-1 in the within sitagliptin group analysis (p = 0.06) and reduced circulating IP-10 and sCD40L in the absence of clinical evidence of systemic inflammation (normal CRP) is even more remarkable since abdominal fat is believed to be a major source of proatherogenic mediators [5, 7]. Thus, it is possible that a strategy of DPP4 inhibition could have beneficial effects on reducing CVD risks via modification of AT inflammation in abdominally obese adults regardless of the presence of systemic markers of inflammation. Randomized clinical trials would be necessary in non-diabetic individuals to test this hypothesis, since results from studies with DPP4 inhibitors in diabetics may not be relevant because that population often already has well established atherosclerosis.

Another clinical trial showed somewhat similar findings to ours [56]. In HIV-positive glucose intolerant patients, who almost universally have systemic inflammation, sitagliptin significantly decreased plasma IP-10 and sCD14 (a marker of monocyte activation) and AT monocyte chemotactic protein-1 (important in facilitating inflammation) compared to placebo. Although sitagliptin decreased biomarkers of inflammation, changes in immune cell phenotypes in AT were not measured in that study.

This pilot study had a number of limitations. The number of participants receiving placebo was quite small and thus the study was underpowered to determine whether more robust effects of DPP4i on immune cells (beyond the changes in ILC-3 and ILC-1) truly occurred, However the significant declines in plasma IP-10 and near significant reductions in sCD40L suggest that the few demonstrable cellular changes in innate lymphocytes are possibly real and attenuation in AT inflammation may have occurred. Yet, we cannot exclude the possibility that the changes were the result of regression to the mean despite the internal consistency that each marker suggested reductions in inflammation. Unexpectedly, the normal plasma CRP suggested that there was little systemic inflammation despite insulin resistance and abdominal obesity.

In this small study, contamination of cell types could have obscured differences. Our gating strategies were based on established gating methodologies [31–33]. For example, Tregs were gated as CD45^+^ CD4^+^ CD127^-^ CD25^Hi^ and Teffs were gated as CD45^+^ CD4^+^ CD127^+^ CD25^+^. This gating strategy yielded respectively more than 75% and 78% purity respectively for Tregs and Teffs as reported by others, suggesting minor contaminations in these populations [31, 32]. If indeed AT is a primary source of adverse cardiometabolic mediators, this tissue should also be evaluated for makers of histologic inflammation, namely for crown-like structures or cellular expression of pro-inflammatory proteins before and after treatment with immune modulators. Finally, our study did not investigate the functional interactions of the changes in the various types of white blood cells. In understanding pathogenesis of AT inflammation, studies should examine whether and how innate and adaptive lymphocytes interact to regulate inflammatory M1 and anti-inflammatory M2 macrophages and monocytes and possibly other immune cells (e.g. NK lymphocytes and stromal cells) and mediators released from adipocytes.

Regardless, a major strength of this study is the demonstration that sufficient quantities of abdominal AT can be obtained that are necessary for innate lymphoid cell typing, unlike lesser quantities needed for quantification of traditional cells of the adaptive immune system because of their greater numbers of the latter cells in tissue. Further, the sizeable amounts of intact tissues that can be obtained with our biopsy procedure maintains histologic integrity so that crown-like structures may also be quantified, and adipocyte size measured with the amount of tissue obtained. Finally, the within group effect sizes generated in this pilot study will be valuable in determining sample sizes for larger, more definitive studies of these biomarkers.

In summary, we have shown that DPP4 inhibition attenuated two cell lines of the innate immune system, one in adipose tissue and one in PBMCs. Reductions in circulating IP-10 and sCD40L suggests that global anti-inflammatory effects may have occurred. Further, in response to our primary study goal, we were able to obtain sufficient abdominal AT with our new biopsy methods to quantify changes in different mononuclear white cells of the innate and adaptive immune systems. Finally, we believe this is the first clinical investigation of the effects of DPP4 inhibition on immune lymphoid cells of innate immunity, namely ILC-1, ILC-2 and ILC-3 cells in AT and blood in obese persons without diabetes. Studies are needed to examine the functional interaction of these different immune cells and their regulatory roles in AT inflammation in individuals with abdominal obesity.

## Supporting information

S1 Checklist CONSORT 2010 checklist– 1.18.2010. (DOC)

S1 Fig Percentage of CD45^+^ cells in peripheral blood mononuclear cells (PBMCs) from participants before and after sitagliptin treatment (A). Percentage of CD45^+^ cells in stromal fraction of adipose tissue from participants before and after sitagliptin treatment (B).
(TIFF)

S2 Fig Frequencies of Tregs/Teffs (A) and M1/M2 (B) in T cell and Monocyte populations respectively in PBMCs from participants before and after sitagliptin treatment. Frequencies of Tregs/Teffs (C) and M1/M2 (D) in T cell and Macrophage populations respectively in stromal fraction of adipose tissue from participants before and after sitagliptin treatment.
(TIFF)

S1 Table Change in absolute counts of immune white cells after 28 days of treatment with sitagliptin. (DOCX)

S2 Table Association of change in glycemic markers with change in ILCs in the sitagliptin group. (DOCX)

S1 File Sattler et al. IRB approved protocol. (DOCX)
