# Supplementary figures and images for: Feasibility of quantifying change in immune white cells in abdominal adipose tissue in response to an immune modulator in clinical obesity
Source: PLoS One. 2020 Sep 3;15(9):e0237496. doi: 10.1371/journal.pone.0237496 (PMC7470412; doi:10.1371/journal.pone.0237496)

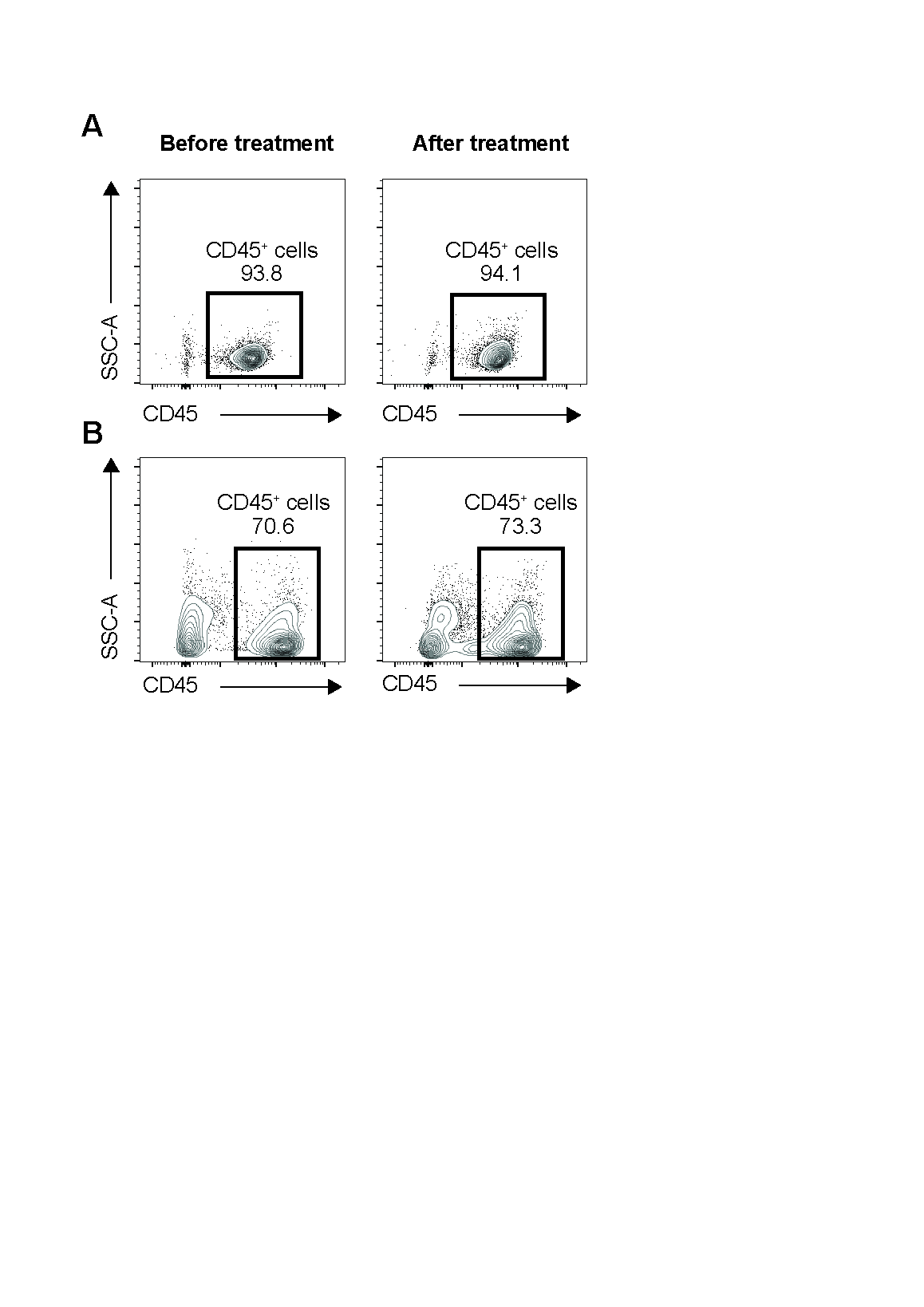

Supplement: S1 Fig — Percentage of CD45+ cells in peripheral blood mononuclear cells (PBMCs) from participants before and after sitagliptin treatment (A). Percentage of CD45+ cells in stromal fraction of adipose tissue from participants before and after sitagliptin treatment (B). (TIFF) [file pone.0237496.s002.tiff]

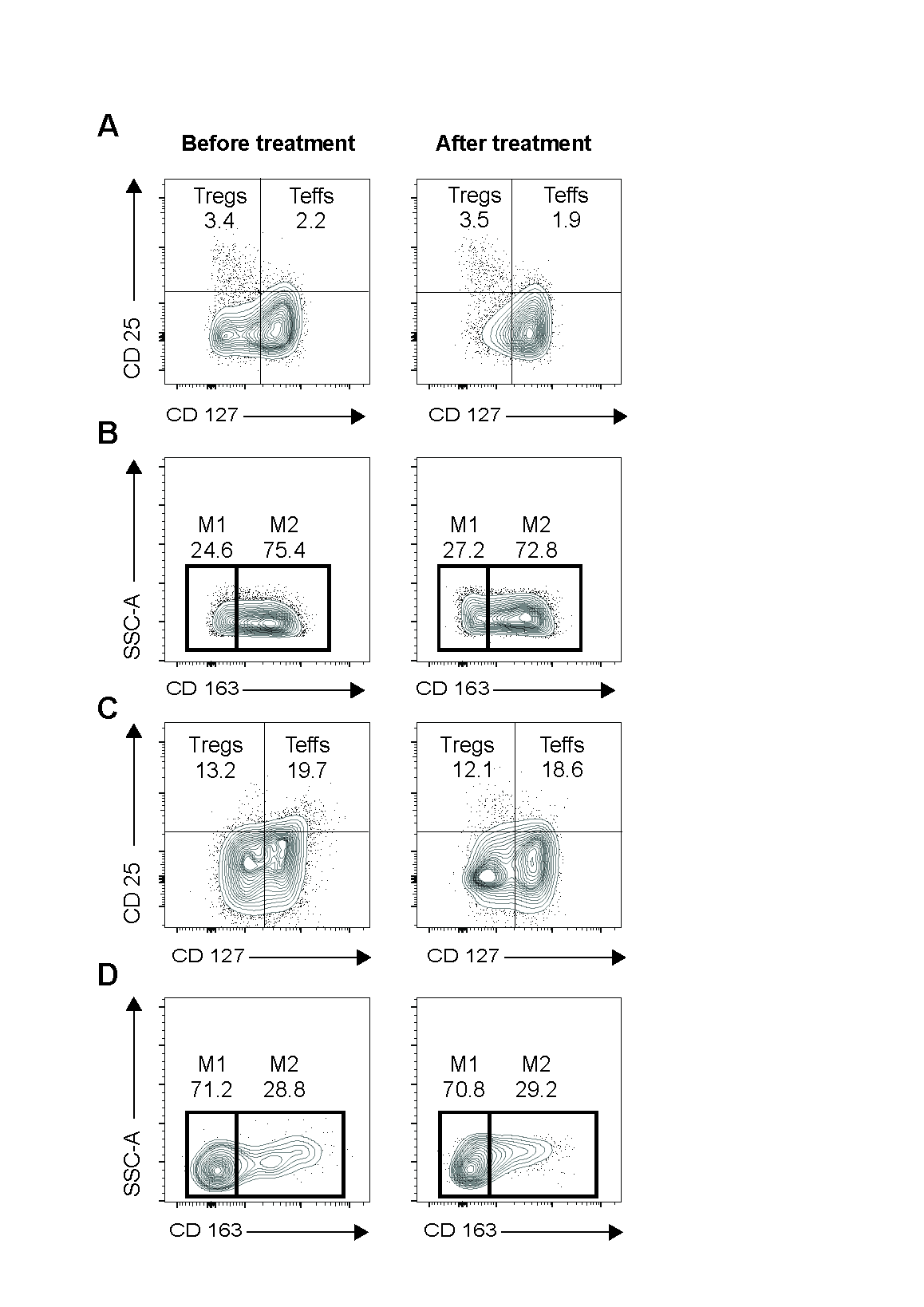

Supplement: S2 Fig — Frequencies of Tregs/Teffs (A) and M1/M2 (B) in T cell and Monocyte populations respectively in PBMCs from participants before and after sitagliptin treatment. Frequencies of Tregs/Teffs (C) and M1/M2 (D) in T cell and Macrophage populations respectively in stromal fraction of adipose tissue from participants before and after sitagliptin treatment. (TIFF) [file pone.0237496.s003.tiff]
