# Supplementary material for: Feasibility of quantifying change in immune white cells in abdominal adipose tissue in response to an immune modulator in clinical obesity
Source: PLoS One. 2020 Sep 3;15(9):e0237496. doi: 10.1371/journal.pone.0237496 (PMC7470412; doi:10.1371/journal.pone.0237496)
Supplement: S1 Table — (DOCX) [file pone.0237496.s004.docx]

**Supplementary Table 1. Change in absolute counts of immune white cells after 28 days of treatment with sitagliptin**

| **Variable (cells)** | **Baseline** | **Day 28** | **Change** | **^#b^P-value** |
| --- | --- | --- | --- | --- |
| **In adipose tissue - absolute counts** (per gram of tissue) | | | | |
| CD45+ | 43920 (33193, 58609)^#a^ | 44689 (34615, 58744) | 32 (-544, 1484) | 0.71 |
| Teffs | 7805 (1869, 10916) | 6416 (1493, 9813) | 291 (-140, 501) | 0.12 |
| Tregs | 3689 (1196, 5319) | 3212 (1107, 5487) | 21 (-321, 141) | 1.00 |
| M1 | 3401 (1443, 7334) | 3693 (1315, 7550) | -49 (-100, 98) | 0.73 |
| M2 | 4073 (1472, 9754) | 4336 (1472, 9642) | 38 (-86, 197) | 0.34 |
| **In PBMCs- absolute counts** (per milliliter of blood) | | | | |
| CD45+ | 1903307 (1237173, 2463853) | 1998861 (1272444, 2350547) | 9290 (-65411, 51227) | 0.95 |
| Teffs | 13871 (8038, 26012) | 14161 (8018, 24292) | -172 (-1579, 25) | 0.09 |
| Tregs | 11420 (7423, 14783) | 11993 (7635, 14103) | 87 (-229, 307) | 0.67 |
| M1 | 2515 (953, 9631) | 2366 (1173, 9277) | 162 (-220, 294) | 0.57 |
| M2 | 8718 (3938, 23541) | 13900 (4794, 33104) | -354 (-1100, --68) | 0.06 |

#a. Values are expressed as median (interquartile range) of the relative percentage of ILCs for baseline, day 28, and change (day 28 – baseline) for all variables (n=14 for both time points)

#b. P values were determined by Wilcoxon signed rank test on change.
