# Supplementary material for: Feasibility of quantifying change in immune white cells in abdominal adipose tissue in response to an immune modulator in clinical obesity
Source: PLoS One. 2020 Sep 3;15(9):e0237496. doi: 10.1371/journal.pone.0237496 (PMC7470412; doi:10.1371/journal.pone.0237496)
Supplement: S2 Table — (DOCX) [file pone.0237496.s005.docx]

|  | **Cell Type** | **N** | **Fasting glucose** | | **Insulin** | | **HOMA-IR** | | **HgbA1C** | |
| --- | --- | --- | --- | --- | --- | --- | --- | --- | --- | --- |
|  |  |  | **r^#a^** | **p-value** | **r** | **p-value** | **r** | **p-value** | **r** | **p-value** |
| ***Counts per ml blood*** | ILC1s | 11 | -0.22 | 0.51 | -0.08 | 0.81 | -0.01 | 0.98 | -0.34 | 0.30 |
|  | ILC2s | 14 | 0.17 | 0.57 | 0.29 | 0.32 | 0.31 | 0.27 | 0.50 | 0.07 |
|  | ILC3s | 11 | -0.42 | 0.19 | -0.16 | 0.63 | -0.13 | 0.71 | 0.30 | 0.38 |
| ***Counts per g adipose tissue*** | ILC1s | 10 | 0.11 | 0.76 | 0.38 | 0.28 | 0.36 | 0.31 | 0.18 | 0.62 |
|  | ILC2s | 13 | 0.11 | 0.73 | 0.04 | 0.89 | 0.16 | 0.60 | 0.39 | 0.19 |
|  | ILC3s | 10 | -0.48 | 0.16 | -0.20 | 0.58 | -0.19 | 0.60 | 0.39 | 0.26 |

**Supplementary Table 2. Association of change in glycemic markers with change in ILCs in the Sitagliptin Group**

#a Spearman correlation coefficients and related p values
