# Supplementary material for: Feasibility of quantifying change in immune white cells in abdominal adipose tissue in response to an immune modulator in clinical obesity
Source: PLoS One. 2020 Sep 3;15(9):e0237496. doi: 10.1371/journal.pone.0237496 (PMC7470412; doi:10.1371/journal.pone.0237496)
Supplement: S1 File — (DOCX) [file pone.0237496.s006.docx]

**Title: Sitagliptin Effects on Arterial Vasoreactivity and Proatherogenic Mediators in Obesity**

**Whittier Foundation Application**

**Principal Investigator: Fred R. Sattler, MD**

Professor of Medicine and Biokinesiology

Department of Medicine, Keck School of Medicine

[fsattler@usc.edu](mailto:fsattler@usc.edu)

2020 Zonal Avenue, IRD Building, Room 434, Los Angeles, 90033

Office Phone: 323-226-4635

**Omid Akbari, PhD**

Associate Professor of Molecular Microbiology & Immunology

Department of Molecular Microbiology & Immunology

akbari@usc.edu

323-442-7930

**Co-Investigators: Howard Hodis, MD**

Professor of Medicine, Preventive Medicine, Molecular Pharmacology and Toxicology

Department of Medicine, Keck School of Medicine

[athero@usc.edu](mailto:athero@usc.edu)

323-442-1478

**Wendy J. Mack, PhD**

Professor of Preventive Medicine

Department of Preventive Medicine

wmack@usc.edu

323-442-1820

**Christina Dieli-Conwright, PhD**

Assistant Professor of Research

Department of Biokinesiology and Physical Therapy

cdieli@usc.edu

323-442-2905

**Abstract**

Almost two-thirds of the US population are obese or overweight, which portends serious health consequences. Indeed, upper body obesity is an independent risk for metabolic syndrome, type II diabetes, and dementia, but mechanisms underlying these relationships are poorly understood. In our prior studies of obesity, we showed that abdominal subcutaneous adipose tissue (aSAT) in almost half of young minority adults is infiltrated with pro-inflammatory macrophages (M1) that is associated with a proatherogenic profile and insulin resistance. We hypothesize that fat inflammation and its associated metabolic dysregulation can be reduced using drugs targeting M1 macrophages and that attenuation of the inflammatory process is associated with reductions in systemic markers of immune activation, pro-atherogenic mediators, and improvements in brachial artery flow-mediated dilation (FMD) and carotid artery elasticity, both sensitive markers of atherosclerosis and cardiovascular risks. We will utilize our novel percutaneous biopsy method to sample deep aSAT, which metabolically and phenotypically more resembles visceral adipose tissue than superficial aSAT, to test our hypotheses. In this study, 20 adults will be randomized (3:1) to treatment with sitagliptin (DPP-4 inhibitor) that down regulates immune activation of monocytes and macrophages versus matching placebo. We will investigate whether sitagliptin significantly reduces M1% in deep aSAT, attenuates systemic proatherogenic mediators (IL-6, TNFα, MCP-1, sICAM-1), markers of immune activation (sCD163, and the M1 signature marker, sCD40L) and whether these changes translate to reductions in FMD and carotid stiffness impairments and improvements in insulin sensitivity. We will also seek to discover a biomarker profile from readily available clinical tests that is highly predictive of inflammatory fat and improvements in FMD and carotid stiffness. Together, these findings are expected to lead to the development of safe and effective therapies targeting tissue macrophage activation that can be used to reduce inflammation in obese persons, including those who are ostensibly healthy, thereby decreasing their risks for developing serious cardiovascular complications, diabetes, and dementia.

**Glossary of Terms**:

aSAT: abdominal subcutaneous adipose tissue

BMI: body mass index

CTSI: Clinical and Translational Sciences Institute

CLS: crown-like structures

hsCRP: highly sensitive C-reactive protein

CVD: cardiovascular disease

DPP-4: dipeptidyl peptidase-4

ESR: erythrocyte sedimentation rate

FACS: fluorescence activated cell sorting

FMD: flow mediated dilation

GLP: glucagon like peptide

HgbA1C: hemoglobin A1C

HOMA-IR: homeostatic method assessment of insulin resistance

ICAM: intracellular adhesion molecule

IR: insulin resistance

M1: pro-inflammatory macrophages

M2: anti-inflammatory macrophages

MCP: monocyte chemoattractant protein

NF-κβ: nuclear factor kappa beta

NSAID: non-steroidal anti-inflammatory drug

Teffector: effector T cells

Tregs: regulatory T-lymphocytes

VAT: visceral adipose tissue

**a. Aims and Hypotheses:** Upper body obesity is a major risk factor for heart attack, stroke, peripheral vascular disease with amputation, Type II diabetes and even dementia. The mechanisms underlying the relationship of obesity to these serious outcomes are poorly understood. In our prior studies of obesity, inflammation with pro-inflammatory macrophages (M1) was present in abdominal subcutaneous adipose tissue (aSAT) biopsies of almost half of the participants, and was associated with insulin resistance (IR) and genomic expression of a pro-inflammatory (e.g. TNFα) and proatherogenic profile[^1^](#_ENREF_1). Indeed, pro-atherogenic mediators such as MCP-1 are released to the systemic circulation and are important for atherogenesis[^2^](#_ENREF_2). Fat inflammation is also linked to systemic markers of monocyte and macrophage activation (sCD163, sCD40L), which are closely associated with IR[^3-5^](#_ENREF_3). Importantly, systemic inflammation and IR are associated with endothelial dysfunction as reflected by circulating vascular adhesion molecules (e.g. sICAM) and impaired brachial artery flow mediated dilatation (FMD)[^6-9^](#_ENREF_6), a sensitive surrogate for atherosclerosis of major vessels and risk for cardiovascular (CV) events[^10-12^](#_ENREF_10). Of note, obese persons without fat inflammation have FMD values similar to lean persons; whereas comparably obese persons with fat inflammation have significant impairments in FMD[^9^](#_ENREF_9). We postulate that inflammation of abdominal fat, when defined by rigorous macrophage phenotyping and quantification using FACS will be closely linked with immune activation markers and tightly associated with abnormal endothelial function, as measured by FMD and carotid artery elasticity. These relationships could provide the basis for macrophage-targeted therapy to reduce obesity-related inflammation. We will use a DPP-4 inhibitor since this class of drugs block signal transduction for macrophage activation[^13-15^](#_ENREF_13). These studies will provide the basis for defining a biomarker profile for the presence of fat inflammation, which can be used to select therapies targeted at macrophage-associated inflammation to reduce CV complications. In obese adults, using methods routinely performed by our team, we will:

**Aim # 1:** Determine if a 4 week course of the DPP-4 inhibitor sitagliptin vs placebo significantly decreases % M1 in deep aSAT and thereby reduces the associated systemic pro-inflammatory and pro-atherogenic state**.**

**Hypothesis 1:** We postulate that DPP-4 inhibition, which reduces monocyte/macrophage activation, will result in repolarization of the M1 to M2 (anti-inflammatory) phenotype in aSAT, thereby decreasing markers of immune activation (sCD163, sCD40L) and outpouring of pro-inflammatory cytokines (IL-6, TNRα) and proatherogenic mediators (MCP-1, sICAM-1) into the systemic circulation**.**

**Aim #2:** Determine if sitagliptin-mediated improvement in adipose inflammation predicts improvement in brachial artery FMD, carotid artery elasticity, and IR compared to participants with less inflammation (lower % M1) in their aSAT.

**Hypothesis 2:** We postulate that the severity of endothelial dysfunction is related to the degree of inflammation in aSAT as assessed by FACS and its associated IR, and participants with increased % M1 macrophages will have greater improvements in FMD, carotid artery elasticity with sitagliptin than placebo.

**Aim #3.** Determine if baseline systemic inflammation and immune activation, abnormal levels of pro-atherogenic mediators, level of insulin resistance and impaired FMD and elasticity can be used to define a biomarker or profile to predict fat inflammation (baseline % M1) and potential response to drug therapy to improve FMD and carotid artery elasticity.

**Hypothesis 3:** Variables examined in this study should enable us to identify a biomarker profile (e.g. hs-CRP, HgbA1C, HOMA-IR, D-dimer, ESR) with high predictive value for fat inflammation, which can be used to screen for patients who have this phenotype and would be candidates for macrophage-targeted therapies.

The importance of this novel study is to demonstrate that targeting macrophage activation by treatment with a DPP-4 inhibitor reduces fat inflammation and attenuate the pro-atherogenic state. Importantly, these effects are expected to improve brachial artery FMD and carotid artery elasticity, two markers of atherosclerosis and serious CV risks. The study will also seek to find a biomarker profile from readily available clinical tests that can be used to identify ostensibly otherwise healthy persons with fat inflammation who would benefit from anti-inflammatory therapy to reduce their subsequent risks for cardiometabolic complications, including heart attack, stroke, peripheral vascular diseases, diabetes, and other serious health conditions. Finally, these outcomes will provide

mechanisms to explain the anticipated CV benefits of 6 large ongoing studies of GLP-1

agonists and DPP-4 inhibitors[^16^](#_ENREF_16).

**b. Background and Significance:** The obesity epidemic is now a global health problem. In the United States, almost 2/3 of American adults (nearly 200 million) are overweight (34.2%) or obese (33.8%)[^17^](#_ENREF_17). Upper body obesity alone increases the risk for heart attack, stroke, peripheral vascular disease and amputation, type II diabetes, and dementia, especially as these people live longer[^18-21^](#_ENREF_18). Mechanisms whereby adiposity causes these serious metabolic-related health co-morbidities remain largely unknown. With cardiovascular disease (CVD), diabetes and dementia, clinical evidence suggests that the risk for these obesity-related conditions is linked to systemic inflammation[^2^](#_ENREF_2)^,^[^18^](#_ENREF_18)^,^[^19^](#_ENREF_19)^,^[^22-24^](#_ENREF_22). In lab animals fed a high fat diet, adipocytes swell and macrophages switch from the “alternative anti-inflammatory” macrophage predominance (M2) to greater numbers of pro-inflammatory macrophages (M1) with outpouring of TNFα, IL-6 and iNOS[^2^](#_ENREF_2)^,^[^25^](#_ENREF_25)^,^[^26^](#_ENREF_26) that promote endothelial dysfunction, IR, atherogenesis, diabetes and CV complications. Crown like structures (CLS, see Figure) due to M1 macrophages around dying fat cells are described with the M1 phenotype[^3^](#_ENREF_3)^,^[^8^](#_ENREF_8). Unfortunately, there is observer variability in deciding whether CLS are present and potential for sampling errors in fat samples. Thus, better methods are needed to determine the presence of fat inflammation and determine the degree of infiltration by M1 cells. Dr. Mittelman’s lab is successfully using FACS to enumerate the actual % of M1 and M2 in fat.

**
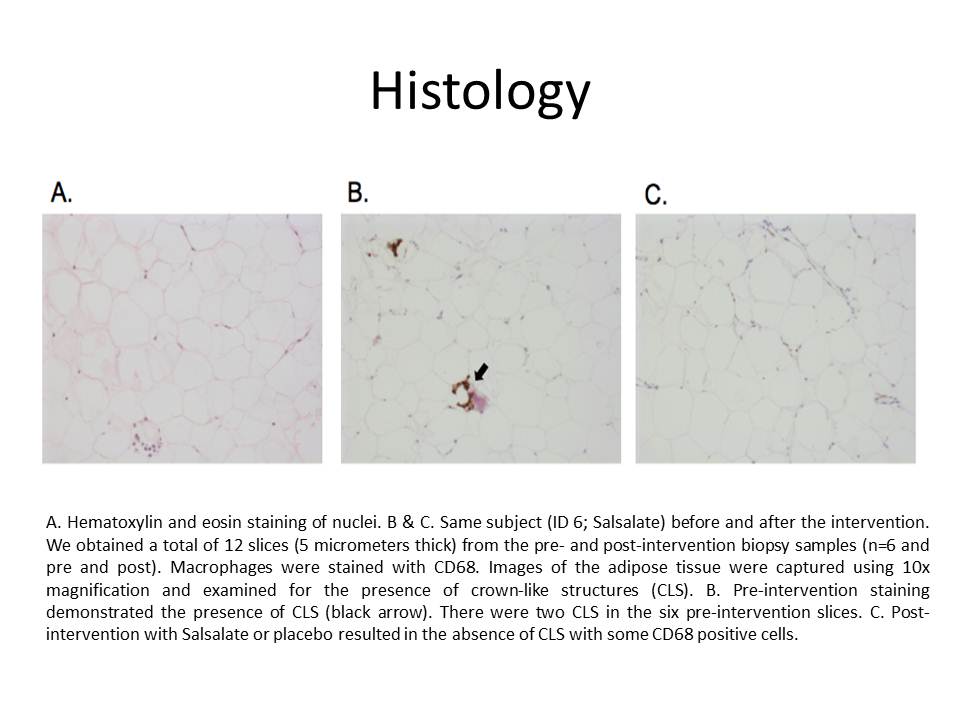
**Two markers of mononuclear cell immune activation appear ideal candidates to assess changes in adipose tissue inflammation. Increased levels of sCD163 are released from activated monocytes/macrophages and are highly correlated with visceral adipose tissue (VAT) and aSAT, IR, elevated IL-6 and TNFα, coronary artery disease (CAD) and inflamed atheroma[^4^](#_ENREF_4)^,^[^27-30^](#_ENREF_27). CD40L is 33kDa transmembrane protein released from activated T-lymphocytes and is pro-inflammatory for endothelial cells and macrophages[^31-34^](#_ENREF_31). sCD40L is also a signature of the M1 phenotype. Obese mice lacking CD40L have less IR and immune cell infiltration in their adipose tissue[^35^](#_ENREF_35). In humans increased levels of sCD40L are associated with inflammatory diseases, myocardial infarction and risk for type II diabetes and its vascular complications[^5^](#_ENREF_5)^,^[^36^](#_ENREF_36)^,^[^37^](#_ENREF_37). Anti-inflammatory treatment of patients with diabetes or CAD reduces their sCD40L levels[^38^](#_ENREF_38)^,^[^39^](#_ENREF_39). Thus, sCD163 and sCD40L appear to be excellent biomarker candidates for macrophage induced aSAT inflammation.

Atherogenesis often takes years to progress to serious clinical complications. Brachial artery flow mediated dilatation (FMD) and carotid artery elasticity have proven to be a sensitive and highly predictive measure of endothelial dysfunction and arterial damage due to atherosclerosis and coronary artery disease, and risk for important CVD events[^10^](#_ENREF_10)^,^[^12^](#_ENREF_12)^,^[^40-42^](#_ENREF_40). Further, abdominal obesity[^7^](#_ENREF_7) and presence of CLS in aSAT has been associated with impairments in FMD[^8^](#_ENREF_8)^,^[^9^](#_ENREF_9). FMD and carotid elasticity are expected to be excellent measures of the effects of obesity on the arterial vascularity, and a means to assess therapy targeted at M1-induced inflammation before vascular changes of atherogenesis become irreversible and progresses to adverse health effects.


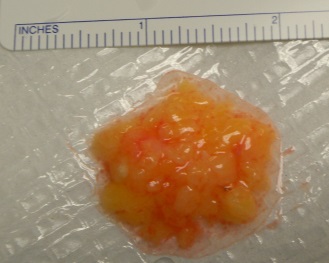
**c. Translational Nature of Project:** In the proposed study, our experienced team of bench-to-bedside researchers takes a novel approach to investigate whether a DPP-4 inhibitor of monocyte/macrophage activation will reduce fat inflammation and its related systemic inflammatory and proatherogenic state. This proposal is feasible based on a new biopsy method developed by Dr. Sattler to obtain greater amounts of aSAT in the range of 2-3g (Figure shows 2.7g), allowing Dr. Akbari’s Lab to precisely sort and enumerate mononuclear white cell fractions by FACS to quantify the degree of M1 infiltration. Importantly, we will examine whether the effects of this therapy can be translated to improvements in FMD and carotid artery elasticity, a highly sensitive measure of atherosclerosis in major arteries and risk for serious CVD events (Dr. Hodis is a world-renowned expert in this methodology). We selected a DPP-4 inhibitor as a probe to investigate the role of immune activation and pro-inflammatory macrophages in adipose tissue in the pathogenesis of serious obesity-related comorbidities. Outcomes of our study can be translated to testing and clinical application of other broad based anti-inflammatory therapies such as NSAIDs and statins to prevent these serious health consequences.

**d. Preliminary Studies:** Dr. Goran’s team reported[^1^](#_ENREF_1) that in aSAT biopsies from obese minority adolescents, there was histologic evidence of inflammation as reflected by CLS in almost half of the participants. Yet, other participants lacked CLS, although the 2 groups had similar clinical features of obesity. Those with CLS had significantly greater IR and elevated TNFα levels, as well as genotypic expression of NF-κB stress pathway activity including the TNF receptor superfamily 11b, LPS binding protein, IL-1ra, MCP-1, and MMP; insulin receptor substrate (IRS)-1 gene expression was down-regulated. In our pilot study of the anti-inflammatory drug salsalate (4g/day) vs placebo for 4 weeks, we obtained deep aSAT biopsies from 15 obese Hispanic adults (18-25 years) before and after treatment. Deep aSAT (below Scarpa’s fascia) is metabolically and phenotypically similar to VAT[^43^](#_ENREF_43)^,^[^44^](#_ENREF_44). There was a trend (Figure) for a decrease in M1% (pro-inflammatory) macrophages (-15.7%, p=0.13) with salsalate that was not observed with placebo (see Figure). This preliminary data “suggests” that salsalate is able to decrease inflammation in aSAT. However, the sample size was too limited to draw definitive conclusions.

**e. Proposed Study,** **Experimental Design and Methods:**

***Design:*** This is a prospective, double-masked, investigation using a 3:1 randomization to sitagliptin (N=15) at the FDA licensed dose of 100mg/day versus matching placebo (N=5) for 28 days in obese adults.


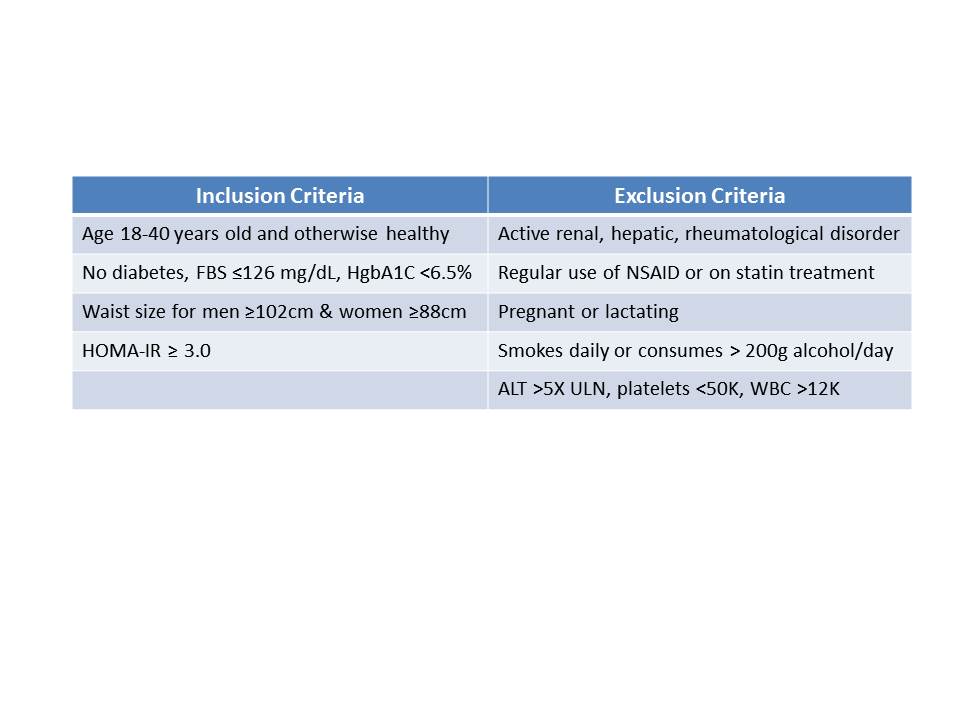


***Eligibility:***

Screening Visits (Visits 1 and 2): Potential study subjects will be screened for eligibility according to the criteria shown in the Table. We chose HOMA-IR ≥3.0, which in our prior studies had a high predictability for increased %M1 in aSAT. Waist circumference (“size”) criteria are those used to define upper body obesity in the metabolic syndrome[^20^](#_ENREF_20). Women will have a pregnancy test done at the second screening visit.

Study Visits:

Visits 4 and 5: Following a 12-hour fast, subjects will be admitted to the USC Clinical Trials Unit for a medical history and physical examination, and superficial SAT biopsy will be obtained.

Visits 3 and 6: Brachial artery FMD and carotid arterial elasticity will be done several days before week 1 and after week 4 visits to assess the relationship of vasoreactivity to fat inflammation and systemic proatherogenic mediators/markers and for changes associated with sitagliptin.

Visits 4 and 5: Blood will be drawn for hsCRP, HbgA1C, D-dimer, and erythrocyte sedimentation rate (ESR) to be tested in the USC Clinical Lab and plasma to be stored at -80C for batch testing later (insulin, glucose, sTNFrII, IL-6, MCP-1, sICAM, sCD40L, and sCD163).

***Methods:*** A novel but important conceptual underpinning of the methodology is that assessing presence of CLS is imprecise due to adipose tissue inflammation heterogeneity, problems with sampling errors, and observer interpretation of what are CLS. We will quantify macrophages in aSAT by FACS for increases in M1%, which we postulate will have a higher predictive value for inflammation than histologic presence of CLS.

We will use ultrasonic guidance to direct biopsies to deep aSAT (below Scarpa’s fascia), which is

phenotypically and metabolically similar to VAT[^43^](#_ENREF_43)^,^[^44^](#_ENREF_44).

Fat Biopsy Procedure: After anesthetizing the skin at the biopsy site (right anterior axillary line, level of the umbilicus), a 6-7mm long skin incision is made. A 6 mm Bergström biopsy needle is introduced through the incision to obtain superficial SAT.. To date, Dr. Sattler has used this method to perform nearly 100 biopsies, yielding an average of 1.5g (often 2-3g) of aSAT per biopsy, which provides more than adequate aSAT for FACS. There have been no adverse events and 85% (17 of 20 participants) completed treatment and both biopsies. Dr. Sattler will do the biopsies.


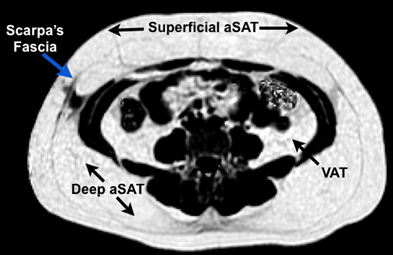


**Figure. Blue arrow shows Scarpa’s fascia. Dark area above and below fascia is site of biopsy several hours earlier.**

Pharmaceuticals: Sitagliptin and the matching placebo will be prepared and dispensed by the USC Investigational Drug Service.

Adherence and Monitoring for Adverse Events (AEs): We will do pill counts to check for medication adherence. Subjects will be called weekly to monitor medication usage and to monitor for AEs. Those with new symptoms will be evaluated by Dr. Sattler and have appropriate blood tests.

Fluorescent Activated Cell Sorting to characterize macro-phages (M) in deep aSAT.and Immunoassays: Fat biopsy samples placed in PBS will be transported on ice and processed within 1-hr for flow cytometry (Dr. Akbari’s lab) to separate M1 and M2 macrophage (Figure below). If additional funding can be procured, cells will be further sorted and enumerated for Tregs, Teffectors, IFNγ-secreting helper T-cells, B-cells, and NK cells, all of which have been shown in animal models to regulate adipose tissue macrophages and inflammation. Banked plasma frozen at -80C will be batch-tested by single platform immunoassays for sTNFrII, IL-6, MCP-1, sICAM, sCD163 and sCD40L.

F**igure:** The left 4 panels show the % of total M for one participant, by gating for CD14+/CD45+ and the % of M1 (CD40+/CD206-) and M2 (CD40/CD206+) cells. Visit 3 shows the changes in M1 and M2 cells after salsalate or placebo (masked). The right 4 panels are for another study subject.

Flow Mediated Dilatation: A high-resolution ultrasound imager equipped with a linear array vascular transducer is used to image the brachial artery in B-mode. Baseline images of the artery and Doppler images of flow are recorded for 15sec. To induce reactive hyperemia, a forearm blood pressure cuff is inﬂated to 250mmHg for 5min and spectral Doppler used to ensure that blood ﬂow ceases. Imaging of the artery begins 15sec prior to deﬂation of the blood pressure cuff and continues for 2min after deﬂation. FMD is calculated as the % change of brachial arterial diameter 1min after cuff deﬂation relative to the baseline [FMD = (P1−P0)/P0×100;

P0 = brachial artery diameter at baseline, P1 = brachial artery diameter 1min after cuff deﬂation].

Carotid Artery Stiffness: The same high-resolution B-mode US with automated computerized edge detection software as described above will be used to determine carotid arterial stiffness, as evaluated by measures of

distensibility and elasticity of the arterial wall (73). Using carotid artery US, measurements of the right common carotid artery diameters at systole (Ds) and diastole (Dd¬) are obtained. Systolic and diastolic blood pressure is measured concomitant with the carotid US to determine the simultaneous pulse pressure (PP). Arterial distensibility and elasticity are calculated as follows:

*Distensibility* = [(2(Ds - Dd)/Dd)/PP] X [10^6^/133.3]

Distensibility index (10^-5^ x N x m^-2^) is a continuous measure of arterial stiffness with lower distensibility indicating greater arterial stiffness.

*Elasticity* (Young’s Elastic Modulus): PP/DD X 0.5 X Dd/CIMTd

Where PP = pulse pressure; DD = % arterial dilation over the cardiac cycle; Dd = arterial diameter at diastole; CIMTd = CIMT at diastole.

Elasticity index (10^-6^ x N^-1^ x m^2^) is a continuous measure of arterial stiffness with higher elasticity index indicating greater arterial stiffness. The coefficients of variation from a repeated-measures study were estimated as 1.8% for CIMT [intra-class correlation coefficient (ICC) = 0.98], 2.2% for CIMTd (ICC = 0.96), and 8.8% for blood pressure (ICC = 0.65–0.73)

*Measures of FMD and carotid elasticity will be done in the Atherosclerosis Research Unit under Direction of Dr. Howard Hodis.*

Statistical Considerations: For Aims #1 & 2, repeated measures ANCOVA will test the between group difference in change across 4 weeks, adjusting for covariates and predictors specified in the aims. For Aim #3, Bootstrap analysis will be used to determine the threshold level of M1% that resulted in significant improvements in FMD, carotid elasticity and IR with sitagliptin. Using this M1% threshold, univariable and multivariable analyses will be used to assess if readily available clinical markers (hs-CRP, HgbA1C, HOMA-IR, D-dimer, ESR) can be used to define a biomarker profile predictive of FMD and carotid elasticity response to anti-inflammatory therapy[^45^](#_ENREF_45). Residual analyses will be performed to determine if assumptions were met, and data transformations made as needed if non-normally distributed data impacts the validity of the results. Effect sizes will be computed for all outcomes to determine the clinical relevance of the findings. Sensitivity analysis was performed using G*Power[^46^](#_ENREF_46) to determine the size of detectible adjusted change in an outcome that would be detectable with the proposed sample size. Assuming up to 10% attrition, α_(two-sided)_ =0.05 and 1-β =0.80, we would be able to statistically detect a moderate within-between group interaction (d=0.52), assuming a moderate between individual correlation (r=0.5). For the group receiving sitagliptin, we would be able to detect a within group change at least d=0.6. This within group change is lower than the effect found in our prior study, which indicated that anti-inflammatory therapy reduced M1% by 16±24% but placebo had no effect (d=0.68). For FMD, the within group change should translate to a detectible difference of at least 7% (using SD of Δ=12%)[^47^](#_ENREF_47). Based on *in vitro* evidence of the direct effects of sitagliptin on macrophage activation, the effect sizes will likely be substantially greater than with the relatively week NSAID (salsalate), thus providing even greater statistical power for the sample size selected. A REDCap database[^48^](#_ENREF_48) will be adapted for this study, allowing secure, web-based data entry, and audit trails for tracking data manipulation and export procedures.

Timeline: We will use the first 2 months to orient staff, develop case report forms, and adapt the REDCap data base to the study. By the end of month 3, we anticipate having CTU approval. During months 3-8, we will screen, recruit and enroll study participants. We expect to complete research collections by the end of the study year, when we begin batch testing specimens. While specimens are processed, we will audit and clean study data and then analyze outcomes with assistance from CTSI statisticians.

**f. Outcomes**: We expect to establish that treatment with a DPP-4 inhibitor will reduce M1 macrophages in abdominal fat and its associated systemic inflammation, insulin resistance and other evidence of a highly proatherogenic state. We expect to show that treatment improves brachial artery FMD and carotid elasticity, both markers of atherosclerosis of major arteries and risk for CVD events. We also anticipate defining a biomarker profile from readily available clinical tests that can be used to identify persons with fat inflammation thereby precluding the need for abdominal fat biopsies prior to treatment. These outcomes, if confirmed in larger studies, could translate to widespread use of anti-inflammatory therapies to improve health by reducing risks for

heart attack, stroke, peripheral artery disease, diabetes, and dementia in obese persons, including otherwise ostensibly healthy people. We will publish our results in high impact journals and apply for additional funding from NIH to further explore mechanisms of macrophage induced fat inflammation and if longer therapy with macrophage specific treatments improves clinical measures of atherosclerosis (e.g. carotid intima media thickness).

**References:**

**1.** Le KA, Mahurkar S, Alderete TL, et al. Subcutaneous adipose tissue macrophage infiltration is associated with hepatic and visceral fat deposition, hyperinsulinemia, and stimulation of NF-kappaB stress pathway. *Diabetes.* Nov 2011;60(11):2802-2809.

**2.** Shoelson SE, Lee J, Goldfine AB. Inflammation and insulin resistance. *J Clin Invest.* Jul 2006;116(7):1793-1801.

**3.** Wentworth JM, Naselli G, Brown WA, et al. Pro-inflammatory CD11c+CD206+ adipose tissue macrophages are associated with insulin resistance in human obesity. *Diabetes.* Jul 2010;59(7):1648-1656.

**4.** Zanni MV, Burdo TH, Makimura H, Williams KC, Grinspoon SK. Relationship between monocyte/macrophage activation marker soluble CD163 and insulin resistance in obese and normal-weight subjects. *Clin Endocrinol (Oxf).* Sep 2012;77(3):385-390.

**5.** Gokulakrishnan K, Deepa R, Mohan V, Gross MD. Soluble P-selectin and CD40L levels in subjects with prediabetes, diabetes mellitus, and metabolic syndrome--the Chennai Urban Rural Epidemiology Study. *Metabolism.* Feb 2006;55(2):237-242.

**6.** Williams IL, Chowienczyk PJ, Wheatcroft SB, et al. Effect of fat distribution on endothelial-dependent and endothelial-independent vasodilatation in healthy humans. *Diabetes Obes Metab.* May 2006;8(3):296-301.

**7.** Arkin JM, Alsdorf R, Bigornia S, et al. Relation of cumulative weight burden to vascular endothelial dysfunction in obesity. *Am J Cardiol.* Jan 1 2008;101(1):98-101.

**8.** Apovian CM, Bigornia S, Mott M, et al. Adipose macrophage infiltration is associated with insulin resistance and vascular endothelial dysfunction in obese subjects. *Arterioscler Thromb Vasc Biol.* Sep 2008;28(9):1654-1659.

**9.** Farb MG, Bigornia S, Mott M, et al. Reduced adipose tissue inflammation represents an intermediate cardiometabolic phenotype in obesity. *J Am Coll Cardiol.* Jul 12 2011;58(3):232-237.

**10.** Widlansky ME, Gokce N, Keaney JF, Jr., Vita JA. The clinical implications of endothelial dysfunction. *J Am Coll Cardiol.* Oct 1 2003;42(7):1149-1160.

**11.** Shechter M, Issachar A, Marai I, et al. Long-term association of brachial artery flow-mediated vasodilation and cardiovascular events in middle-aged subjects with no apparent heart disease. *Int J Cardiol.* May 1 2009;134(1):52-58.

**12.** Davignon J, Ganz P. Role of endothelial dysfunction in atherosclerosis. *Circulation.* Jun 15 2004;109(23 Suppl 1):III27-32.

**13.** Ta NN, Li Y, Schuyler CA, Lopes-Virella MF, Huang Y. DPP-4 (CD26) inhibitor alogliptin inhibits TLR4-mediated ERK activation and ERK-dependent MMP-1 expression by U937 histiocytes. *Atherosclerosis.* Dec 2010;213(2):429-435.

**14.** Makdissi A, Ghanim H, Vora M, et al. Sitagliptin exerts an antinflammatory action. *J Clin Endocrinol Metab.* Sep 2012;97(9):3333-3341.

**15.** Chaudhuri A, Ghanim H, Vora M, et al. Exenatide exerts a potent antiinflammatory effect. *J Clin Endocrinol Metab.* Jan 2012;97(1):198-207.

**16.** Nauck MA. A Critical Analysis of the Clinical Use of Incretin-Based Therapies: The benefits by far outweigh the potential risks. *Diabetes Care.* May 6 2013.

**17.** Flegal KM, Carroll MD, Kit BK, Ogden CL. Prevalence of obesity and trends in the distribution of body mass index among US adults, 1999-2010. *Jama.* Feb 1 2012;307(5):491-497.

**18.** Pou KM, Massaro JM, Hoffmann U, et al. Visceral and subcutaneous adipose tissue volumes are cross-sectionally related to markers of inflammation and oxidative stress: the Framingham Heart Study. *Circulation.* Sep 11 2007;116(11):1234-1241.

**19.** Panza F, Frisardi V, Seripa D, et al. Metabolic syndrome, mild cognitive impairment, and dementia. *Curr*

*Alzheimer Res.* Aug 2011;8(5):492-509.

**20.** Grundy SM, Cleeman JI, Daniels SR, et al. Diagnosis and management of the metabolic syndrome: an American Heart Association/National Heart, Lung, and Blood Institute Scientific Statement. *Circulation.* Oct 25 2005;112(17):2735-2752.

**21.** Sparano JA, Wang M, Zhao F, et al. Obesity at diagnosis is associated with inferior outcomes in hormone receptor-positive operable breast cancer. *Cancer.* Dec 1 2012;118(23):5937-5946.

**22.** Yaffe K, Kanaya A, Lindquist K, et al. The metabolic syndrome, inflammation, and risk of cognitive decline. *Jama.* Nov 10 2004;292(18):2237-2242.

**23.** Goldfine AB, Fonseca V, Jablonski KA, Pyle L, Staten MA, Shoelson SE. The effects of salsalate on glycemic control in patients with type 2 diabetes: a randomized trial. *Ann Intern Med.* Mar 16 2010;152(6):346-357.

**24.** Hotamisligil GS. Inflammation and metabolic disorders. *Nature.* Dec 14 2006;444(7121):860-867.

**25.** Lumeng CN, Deyoung SM, Bodzin JL, Saltiel AR. Increased inflammatory properties of adipose tissue macrophages recruited during diet-induced obesity. *Diabetes.* Jan 2007;56(1):16-23.

**26.** Lumeng CN, DelProposto JB, Westcott DJ, Saltiel AR. Phenotypic switching of adipose tissue macrophages with obesity is generated by spatiotemporal differences in macrophage subtypes. *Diabetes.* Dec 2008;57(12):3239-3246.

**27.** Aristoteli LP, Moller HJ, Bailey B, Moestrup SK, Kritharides L. The monocytic lineage specific soluble CD163 is a plasma marker of coronary atherosclerosis. *Atherosclerosis.* Feb 2006;184(2):342-347.

**28.** Moreno JA, Munoz-Garcia B, Martin-Ventura JL, et al. The CD163-expressing macrophages recognize and internalize TWEAK: potential consequences in atherosclerosis. *Atherosclerosis.* Nov 2009;207(1):103-110.

**29.** Subramanian S, Tawakol A, Burdo TH, et al. Arterial inflammation in patients with HIV. *Jama.* Jul 25 2012;308(4):379-386.

**30.** Parkner T, Sorensen LP, Nielsen AR, et al. Soluble CD163: a biomarker linking macrophages and insulin resistance. *Diabetologia.* Jun 2012;55(6):1856-1862.

**31.** Salemi J, Obregon DF, Cobb A, et al. Flipping the switches: CD40 and CD45 modulation of microglial activation states in HIV associated dementia (HAD). *Mol Neurodegener.* 2011;6(1):3.

**32.** Mach F, Schonbeck U, Sukhova GK, et al. Functional CD40 ligand is expressed on human vascular endothelial cells, smooth muscle cells, and macrophages: implications for CD40-CD40 ligand signaling in atherosclerosis. *Proc Natl Acad Sci U S A.* Mar 4 1997;94(5):1931-1936.

**33.** Mach F, Schonbeck U, Bonnefoy JY, Pober JS, Libby P. Activation of monocyte/macrophage functions related to acute atheroma complication by ligation of CD40: induction of collagenase, stromelysin, and tissue factor. *Circulation.* Jul 15 1997;96(2):396-399.

**34.** Seijkens T, Kusters P, Engel D, Lutgens E. CD40-CD40L: linking pancreatic, adipose tissue and vascular inflammation in type 2 diabetes and its complications. *Diab Vasc Dis Res.* Mar 2013;10(2):115-122.

**35.** Poggi M, Engel D, Christ A, et al. CD40L deficiency ameliorates adipose tissue inflammation and metabolic manifestations of obesity in mice. *Arterioscler Thromb Vasc Biol.* Oct 2011;31(10):2251-2260.

**36.** Varo N, Libby P, Nuzzo R, Italiano J, Doria A, Schonbeck U. Elevated release of sCD40L from platelets of diabetic patients by thrombin, glucose and advanced glycation end products. *Diab Vasc Dis Res.* May 2005;2(2):81-87.

**37.** Devaraj S, Glaser N, Griffen S, Wang-Polagruto J, Miguelino E, Jialal I. Increased monocytic activity and biomarkers of inflammation in patients with type 1 diabetes. *Diabetes.* Mar 2006;55(3):774-779.

**38.** Marx N, Imhof A, Froehlich J, et al. Effect of rosiglitazone treatment on soluble CD40L in patients with type 2 diabetes and coronary artery disease. *Circulation.* Apr 22 2003;107(15):1954-1957.

**39.** Heeschen C, Dimmeler S, Hamm CW, et al. Soluble CD40 ligand in acute coronary syndromes. *N Engl J Med.* Mar 20 2003;348(12):1104-1111.

**40.** Selzer RH, Mack WJ, Lee PL, Kwong-Fu H, Hodis HN. Improved common carotid elasticity and intima-media thickness measurements from computer analysis of sequential ultrasound frames. *Atherosclerosis.* Jan 2001;154(1):185-193.

**41.** Mack WJ, LaBree L, Liu C, Selzer RH, Hodis HN. Correlations between measures of atherosclerosis change using carotid ultrasonography and coronary angiography. *Atherosclerosis.* Jun 2000;150(2):371-379.

**42.** Karim R, Mack WJ, Kono N, et al. T-cell activation, both pre- and post-HAART levels, correlates with carotid artery stiffness over 6.5 years among HIV-infected women in the WIHS. *J Acquir Immune Defic Syndr.* Nov 1 2014;67(3):349-356.

**43.** Kelley DE, Thaete FL, Troost F, Huwe T, Goodpaster BH. Subdivisions of subcutaneous abdominal adipose tissue and insulin resistance. *Am J Physiol Endocrinol Metab.* May 2000;278(5):E941-948.

**44.** Tordjman J, Divoux A, Prifti E, et al. Structural and inflammatory heterogeneity in subcutaneous adipose tissue: relation with liver histopathology in morbid obesity. *J Hepatol.* May 2012;56(5):1152-1158.

**45.** Faul F, Erdfelder E, Buchner A, Lang AG. Statistical power analyses using G*Power 3.1: tests for correlation and regression analyses. *Behav Res Methods.* Nov 2009;41(4):1149-1160.

**46.** Faul F, Erdfelder E, Lang AG, Buchner A. G*Power 3: a flexible statistical power analysis program for the social, behavioral, and biomedical sciences. *Behav Res Methods.* May 2007;39(2):175-191.

**47.** Shechter M, Sharir M, Labrador MJ, Forrester J, Silver B, Bairey Merz CN. Oral magnesium therapy improves endothelial function in patients with coronary artery disease. *Circulation.* Nov 7 2000;102(19):2353-2358.

**48.** Harris PA, Taylor R, Thielke R, Payne J, Gonzalez N, Conde JG. Research electronic data capture (REDCap)--a metadata-driven methodology and workflow process for providing translational research informatics support. *J Biomed Inform.* Apr 2009;42(2):377-381.
